# Electrode Engineering for High‐Durability Variable‐Emissivity Devices Based on Reversible Copper Electrodeposition

**DOI:** 10.1002/advs.202520497

**Published:** 2025-12-23

**Authors:** Runyun He, Tianwen Liu, Liqiang Zhang, Tuoyu Liu, Dan Tang, Wenxia Zhang, Jundong Tao, Tingting Shi, Yijing Song, Haifeng Cheng, Yao Zhang, Dongqing Liu

**Affiliations:** ^1^ Science and Technology on Advanced Ceramic Fibers and Composites Laboratory College of Aerospace Science and Engineering National University of Defense Technology Changsha 410073 P. R. China; ^2^ China Academy of Aerospace System and Innovation Beijing 100088 P. R. China

**Keywords:** cycling performance, reversible copper electrodeposition, ultra‐thin metal electrode, variable emissivity devices

## Abstract

Variable emissivity devices enable dynamic infrared emissivity modulation for thermal camouflage, energy‐efficient architecture, spacecraft thermal control, and wearable thermoregulators. While reversible metal electrodeposition allows remarkable spectral modulation via controlled metal deposition/dissolution, electrode degradation‐induced cycling instability hinders practical application. Ultra‐thin Ir‐based electrode strategy is developed to enhance durability of reversible copper electrodeposition variable‐emissivity device. The Ir‐electrode‐enabled devices exhibit merely 11% degradation in radiative temperature variation after 8000 cycles and 23% attenuation in emissivity modulation after 6000 cycles, substantially outperforming Pt‐based counterparts (54% attenuation after 5000 cycles). This stability stems from Ir's inherent chemical inertness, superior conductivity, and mechanical robustness, which collectively suppress stress‐induced cracking, electrolyte corrosion, and electrochemical oxidation. Ir‐Au grid composite electrode ensures homogeneous deposition/dissolution, maintaining performance in large‐area rigid and flexible devices (>3000 cycles on 10 × 10 cm^2^ rigid Si, >1000 cycles on 8 × 8 cm^2^ flexible polyamide). Integration of Cr_2_O_3_ optical interference layer with Ir electrode on BaF_2_ enables dual‐band modulation (visible coloration and 3–14 µm infrared wavelength, emissivity modulation range Δε = 0.56) with stability exceeding 10 000 cycles. The Ir‐based electrode engineering exhibits enhanced stability, scalable manufacturability, compliant substrate integration, and color‐compatible operation, providing pivotal implications for thermal regulation systems, IR stealth applications, and advanced display technology.

## Graphical abstract

An Ir‐based reversible copper electrodeposition variable‐emissivity device achieved over 8000 cycles with just 11% radiative temperature variation decay. This stability stems from Ir's inherent inertness, high conductivity, and strength, which collectively suppress cracking and corrosion. The electrode design offers scalable production, flexibility, and color compatibility for thermal management and infrared stealth applications.

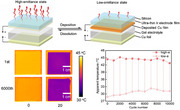

## 1 Introduction

Variable emissivity devices represent an emerging class of optoelectronic systems that enable real‐time modulation of infrared thermal emissivity. These technologies offer transformative potential across a range of applications, including thermal camouflage for defense, dynamic thermal management in energy‐efficient buildings, precision thermal control for spacecraft, and personal thermal regulation in advanced wearable devices. Among the various actuation mechanisms, reversible metal electrodeposition (RME) has emerged as a particularly promising approach due to its ability to achieve broad‐spectrum modulation across infrared wavelengths through the controlled electrochemical deposition and dissolution of metal layers. This capability allows for desirable infrared tunability, making RME‐based devices a leading candidate for next‐generation emissivity control. Recently, multiple metallic systems, such as Ag,^[^ ^1^, ^2^ ^]^ Bi,^[^ ^1^, ^3^, ^4^, ^5^, ^6^ ^]^ Cu,^[^ ^7^, ^8^, ^9^ ^]^ Ni,^[^ ^10^, ^11^ ^]^ Zn,^[^ ^12^, ^13^, ^14^ ^]^ and Pb^[^ ^15^, ^16^, ^17^, ^18^ ^]^ are explored to spectral modulation through electrochemical manipulation, enabling multifunctional optoelectronic integration.^[^ ^19^ ^]^ Our earlier work established that leveraging localized surface plasmon resonance (LSPR) in ultrathin metal electrodes enables superior infrared emissivity modulation via reversible Ag metal electrodeposition.^[^ ^20^ ^]^ The resulting devices have large, uniform, and consistent infrared (IR) tunability in both mid‐wave IR (3 to 5 µm) and long‐wave IR (7.5 to 13 µm) atmospheric transmission windows (emissivity modulation range Δε_MWIR_ ≈ 0.77; Δε_LWIR_ ≈ 0.71). These advances have demonstrated that RME holds significant promise for a wide range of high‐end applications.

However, the practical deployment of such systems faces a significant challenge: poor cycling stability caused by gradual electrode degradation. Recent reports on stability performances of reversible metal electrodeposition devices for infrared emissivity control (benchmarks in Table S1, Supporting Information^[^ ^20^, ^21^, ^22^, ^23^, ^24^, ^25^, ^26^, ^27^, ^28^ ^]^) reveals that most devices maintain cycling stability below 2000 cycles, constituting a critical impediment to practical applications. The origin stems from the repeated redox cycling on surface of electrodes, which leads to morphological changes, loss of electrical contact, and eventual failure of the electrodeposited layer, thereby limiting the long‐term operational viability and reliability. Therefore, overcoming these durability barriers remains a critical hurdle toward commercialization and broader adoption.

To overcome stability limitations, advanced approaches have emerged, including voltage optimization, electrolyte selection and electrode optimization. For voltage optimization, pulsed protocols with periodic maintenance cycles (e.g., multistep cyclic voltammetry) effectively remove residual metal deposits and extend device lifespan.^[^ ^29^ ^]^ However, these methods proved difficult to control and demonstrated limited efficacy. In terms of electrolyte selection, introducing transition metal ions,^[^ ^10^ ^]^ polymer networks,^[^ ^30^ ^]^ poly (ionic liquid)s,^[^ ^31^ ^]^ or redox mediators.^[^ ^32^ ^]^ enhances structural stability, but certain additives induce parasitic reactions, necessitating precise compositional balance.^[^ ^23^ ^]^ From the perspective of electrode optimization, electrode engineering employs nanoparticle modification and superstructured electrodes to mitigate cycling‐induced lattice stress;^[^ ^18^, ^21^ ^]^ doped electrodes improve surface conductivity and reduce interfacial Schottky barrier height;^[^ ^33^, ^34^ ^]^ porous or mesh electrodes (e.g., graphene) overcome ion transport constraints and volumetric expansion in dense electrodes;^[^ ^22^, ^24^ ^]^ protective coatings isolate electrodes from electrolytes, suppressing corrosion and byproduct formation.^[^ ^30^ ^]^ These synergistic strategies offer viable solutions and improve the cycling stability, while the scaling for large‐scale devices, especially the flexible devices, remains challenging. Consequently, simpler and more efficient methods are imperative to achieve the long‐term cycling stability essential for practical reversible metal electrodeposition device  (RMED) applications.

Building on these foundations, we developed an innovative electrode design strategy to significantly enhance the cycling durability of copper‐based RMED devices for infrared emissivity modulation. Iridium—a platinum‐group metal—was selected due to its exceptional electrochemical stability under corrosive and oxidizing environments, wide voltage‐windows, and mechanical resilience—including resistance to stress‐induced cracking, delamination, and corrosion, all essential for long‐term device viability. Crucially, lattice matching with Cu film ensures high compatibility with deposited metal layers to enable uniform and dense electrodeposition. The Ir‐electrode‐enabled devices display significantly enhanced cycling stability, with radiative temperature variation showing only an 11% degradation (from 5.7 to 5.1 °C) after 8000 cycles and 23% attenuation in emissivity modulation capability after 6000 cycles (54% attenuation in emissivity modulation capability of Pt‐electrode‐based devices after 5000 cycles). To demonstrate the feasibility and generality of Ir electrode for practical devices, we develop Ir/Au composite metallic grids to improve current distribution over large areas, and integrating these designs with both rigid and flexible substrates. As a result, we achieved over 3000 cycles in 10×10 cm^2^ Si‐based devices and over 1000 cycles in 8×8 cm^2^ flexible biaxially oriented polyamide film (BOPA)‐based devices. Furthermore, by combining Ir electrodes with Cr_2_O_3_ optical layers on BaF_2_ substrate, we constructed dual‐band modulation devices capable of simultaneous visible coloration and mid‐far infrared emissivity control (Δε = 0.56) for more than 10000 cycles. This study conclusively demonstrates that rational electrode design is pivotal to advancing the cycling performance and real‐world applicability of RMED technology.

## 2 Results and Discussion

### 2.1 Cycling Instability in Reversible Copper Electrodeposition Devices

**Figure 1:**
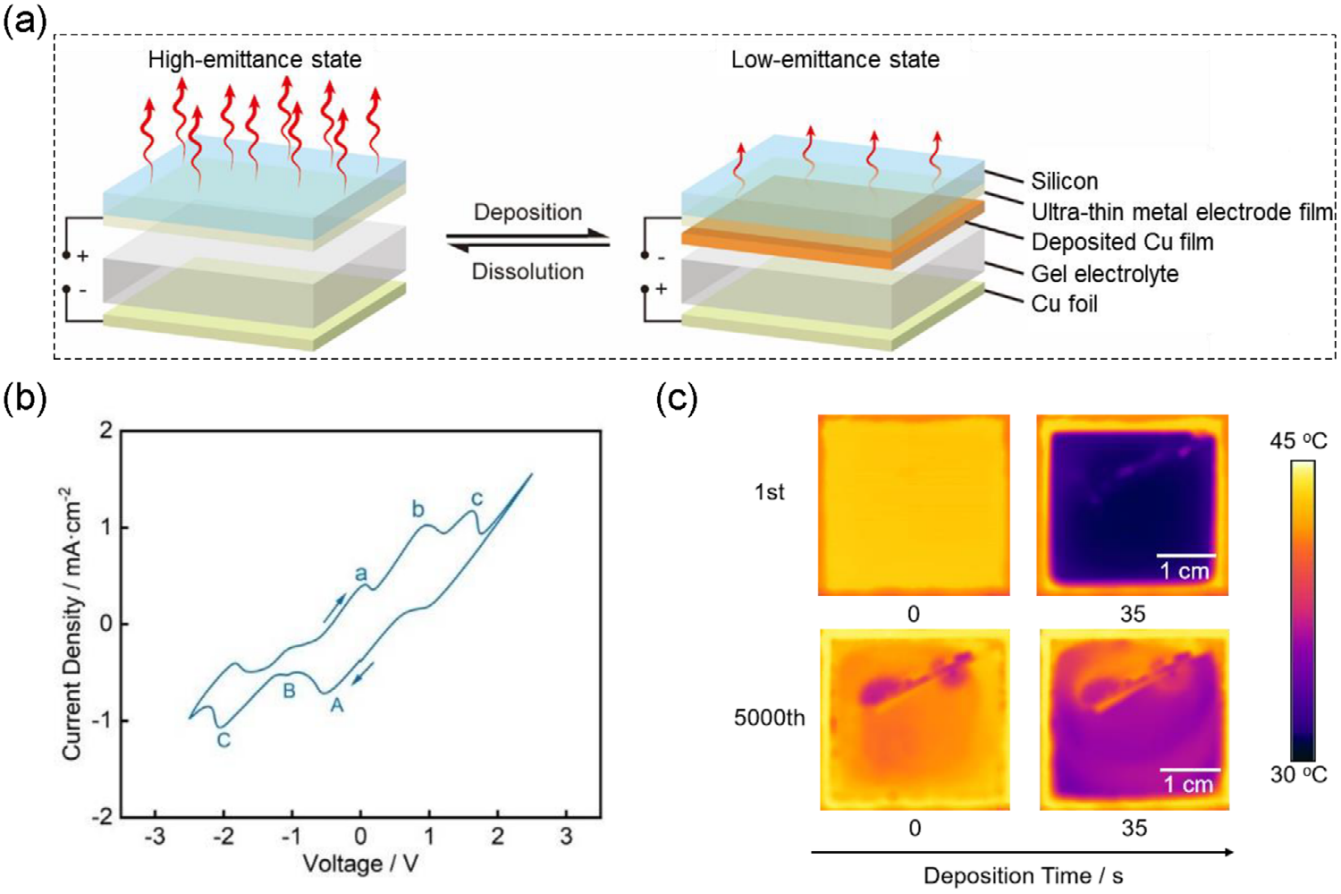
Working mechanism and cycling instability in reversible copper electrodeposition devices. a) Schematic diagram of dynamic emissivity modulation mechanism in copper reversible electrodeposition device. b) Cyclic voltammetry characteristics of copper‐based device with ultrathin Pt electrode on Si substrate (3 × 3 cm^2^). c) cycling performances probed by real‐time IR images of copper‐based device with ultrathin Pt electrode on Si substrate (3 × 3 cm^2^) during the cycling test.

**Figure** **1**a illustrates the structure of reversible copper electrodeposition variable emissivity device. The device (3 × 3 cm^2^) comprises Pt working electrode (on Si substrate, Si substrate IR transmittance (0.55 in 3–5 µm, 0.50 in 8–14 µm), Figure S1; Table S2, Supporting Information), copper foil counter electrode, and intervening gel electrolyte. Nanostructured Pt working electrode has three key roles: (1) Strong electron scattering in nanoscale Pt films induces high optical losses, (2) Incomplete electromagnetic wave shielding due to insufficient free‐electron density in ultrathin Pt enables IR transmission, (3) Reversible metal electrodeposition mediates dynamic IR absorption‐transmission‐reflection conversion. Consequently, the reversible metal electrodeposition devices integrating nanoscale Pt films achieve cyclable transitions between high‐ and low‐emissivity states.^[^ ^20^ ^]^ In the initial state without copper deposition, the device exhibits high emissivity characteristics due to high absorption of electrolyte and ultrathin metal. Cyclic voltammetry measurements (±2.5 V) in two‐electrode configuration revealed three distinct redox processes (Figure 1b): Cu^2+^→Cu^+^ reduction at −0.5 V (peak A), Cu^+^→Cu^0^ deposition at −1 V (peak B), and direct Cu^2+^→Cu^0^ reduction at higher voltages (peak C). This demonstrates that optimal copper deposition occurs between −0.5 to −1.0 V, so increase deposition voltage (more than −1.0 V) can not only increase the current density, but also Cu^2+^ can be directly reduced to Cu (reduction peak C), enabling emissivity regulation at a faster rate. Dissolution process exhibited fundamentally distinct characteristics. Cu layer underwent complete dissolution at voltage as low as 0.5 V, a phenomenon attributed to enhanced dissolution kinetics resulting from spontaneous redox reaction between Cu^2+^ and Cu^0^. The persistent current observed after complete dissolution suggested occurrence of halogen ion side reactions, the impact of which on device longevity required validation through further cycling tests.

Upon application of a deposition voltage, the grown Cu film on the traditional ultra‐thin Pt electrode transitions from being infrared‐transparent to reflective behavior, effectively suppressing infrared radiation, thus forming low emissivity state. The real‐time IR thermal imaging characterization at 50 °C demonstrates significant radiation temperature evolution under cycling process (Figure 1c): the initial high‐emissivity state registers 42.5 °C, following a 35‐s deposition at −2.5 V, the surface temperature of the low‐emissivity coating reaches 31.7 °C, yielding a remarkable 10.8 °C regulation range. The cycling life is defined as the number of cycles before overall radiative temperature change of device decreases to less than 1 °C in this study. Correspondingly, as shown in Figure S2 (Supporting Information), Initial cycles exhibited exceptional performance with Δε = 0.41 (3–14 µm) and 120% Coulombic efficiency, attributable to the comproportionation reaction between Cu^2+^ and metallic Cu. After 5000 cycles, the device maintained 95% Coulombic efficiency with Δε = 0.19 (54% decay) and 5.8 °C radiation temperature modulation (46% decay). The devices with Pt electrode suffer from a significant decay in its emissivity modulation range during cycling, which stem from the accumulation of oxides and inactive metal residues on its surface during cycling. This is evidenced by scanning electron microscope (SEM) images (Figure S3, Supporting Information) and Energy dispersive spectroscopy (EDS) results of the electrode after copper deposition (Figure S4, Supporting Information), demonstrating that the deposited metal film shows discontinuous morphology and residue accumulation on the electrode surface after cycling, suggesting significant loss of electrochemical activity or even localized electrode failure. These deposits not only impede charge transfer, metal ion accessibility and diminish electrochemical activity, but also give rise to local nonuniform electric fields at electrode‐electrolyte interface, thereby leading to nonuniform metal deposition and metal dendrite growth.^[^ ^35^ ^]^ Furthermore, inactive/isolated metal residues on electrode surface increase the initial reflectance of working electrode, causing a substantial decline in device's emissivity modulation performance and ultimately triggering device failure.

### 2.2 The Effect of Ultra‐Thin Metal Electrodes on Cycling Performance

To investigate the impact of metal electrodes on the device cycling performance, we comprehensively analyzed key parameters, including the standard reduction potential, resistivity, thermal expansion coefficient and crystal structure and associated constants. The standard reduction potential is the most critical factor, as the reversible metal electrodeposition in variable‐emissivity devices relies on metal redox reactions. For stable operation, the ultra‐thin metal electrodes must be more inert than the deposited metal, requiring a more positive standard reduction potential and maintaining electrochemical stability under positive bias. Table S3 (Supporting Information) presents the physicochemical properties of metals (4 nm‐thick Pt, Ir, Cr, and Au on silicon wafers, with a 1 nm Cr adhesion layer for Au), ordered by their standard reduction potentials (using aqueous solution values, with potential deviations expected in DMSO‐based electrolytes). Electrochemical testing (Figure S5, Supporting Information) revealed distinct behaviors: the Cr electrode dissolved at elevated voltages, Au showed insufficient current density for practical response speeds, and Pt experienced performance degradation due to chloride ion adsorption. In contrast, the Ir electrode demonstrated superior stability, maintaining consistent diffusion current over an extended period. This stability, combined with its favorable lattice match (face‐centered cubic structure with minimal lattice mismatch to Cu), positions Ir as the optimal electrode material for sustained cycling performance and high‐quality Cu electrodeposition.

**Figure 2:**
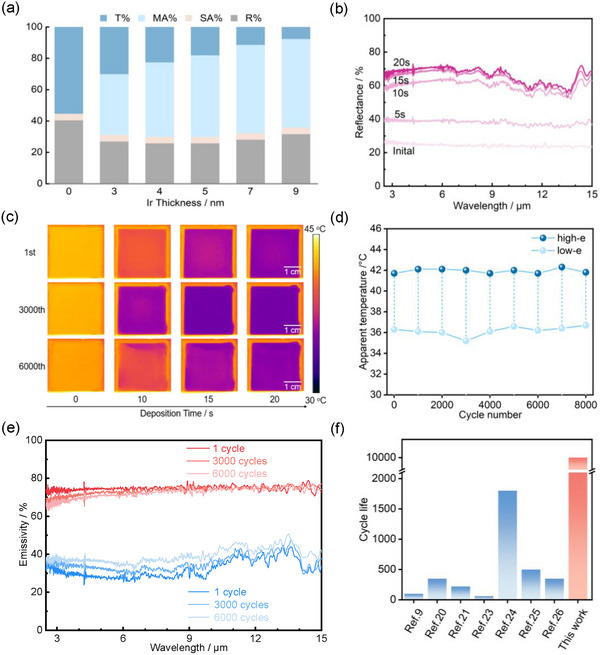
The effect of ultra‐thin Ir metal electrodes on cycling performance of reversible copper electrodeposition devices. a) IR response of the Ir‐Si electrode with different Ir thickness, where T% = transmittance, MA% = metal‐induced absorbance, SA% = substrate‐induced absorbance, R% = reflectance. The theoretical maximum emittance change of a Ir‐Si electrode is equal to the sum of T% and MA%, which are able to be transferred to reflectance by Cu electrodeposition. b) IR reflectance changes of the device at different electrodeposition times. c) Real‐time IR images of the device during the cycling test. d) Radiative temperature variation of the device during cycling. e) IR emissivity changes of the device at different cycle numbers. Figures c, d, and e all show the cycling performance of reversible copper electrodeposition device fabricated with ultra‐thin Ir electrode on Si substrate (3 × 3 cm^2^). f) Comparison of cycling performance of dual‐band compatible Cu electrodeposition device based on ultra‐thin Ir electrode on BaF_2_ substrate (3 × 3cm^2^) with other RMEDs reported to date.

Nanoscale metal films exhibit infrared transmittance due to incomplete electromagnetic shielding from limited free electron density. Concurrently, evaporated Ir films on heterogeneous surfaces form nanoscale clusters, with boundary electron scattering enabling infrared absorption. **Figure** **2**a correlates Ir thickness with optical properties (T% = transmittance, MA% = Ir absorption, SA% = Si absorption, R% = reflectivity). The bare Si substrate (0 nm Ir) exhibits 55.4% T% and 4.2% SA% (3–14 µm), both infrared absorption and transmission of Ir can be converted into infrared reflection through metal electrodeposition, thus the achievable infrared modulation range equals sum of infrared absorption and transmission, theoretically permitting Δε > 0.5 with Cu electrodeposition. Furthermore, the reflectance of Ir electrode evaporated on Si substrate initially decreases and then increases with film thickness, reaching its minimum at 4 nm. This suggests that the optimal thickness of Ir as an electrode material for maximizing the theoretical emissivity modulation range is 4 nm. The reversible copper electrodeposition device incorporating 4 nm Ir electrode demonstrates significant emissivity modulation range (Δε = 0.43) with rapid response characteristics, reaching maximum emissivity change within 15 s. As shown in Figure 2b, the device's reflectance stabilizes after 20 s, indicating minimal further optical change despite continued Cu deposition. Figure S6 (Supporting Information) reveals distinct two‐stage Cu deposition kinetics. The nucleation stage within initial 10 s shows modest reflectance change (7%), followed by a rapid growth phase where reflectance increased by 28% in the subsequent 10 s. This fourfold acceleration in optical response after nucleation completion demonstrates the crucial transition from nucleation‐limited to diffusion‐controlled growth regimes. Compared to devices with Pt electrode, the device configuration reduces each cycle's duration by 40 s, significantly improving the modulation efficiency, which may be due to the higher reactivity of Cu layer on the Ir electrode surface.

The reversible copper electrodeposition device fabricated with ultra‐thin Pt electrode on Si substrate (3 × 3 cm^2^) placed on heating stage (50 °C, constant‐temperature conditions) was subjected to ±2.5 V cycling with 20 s polarity switching (40 s/cycle), infrared thermal imaging (Figure 2c) and radiative temperature profiles (Figure 2d) revealed the evolution of surface temperature during cycling. The device demonstrated exceptional cycling stability, maintaining stable emissivity modulation over 6000 cycles without any functional degradation. This performance is attributed to a highly reversible deposition‐dissolution process, which exhibited no short‐circuiting, open‐circuit phenomena, or reduction in effective deposition area. The device exhibited excellent stability, with its radiative temperature variation showing only an 11% degradation (from 5.7 to 5.1 °C) after 8000 cycles. This minimal attenuation confirms its sustained capability for dynamic emissivity modulation. Residual metallic particles during cycling act as permanent nucleation sites, thereby accelerating subsequent deposition rates and enhancing modulation kinetics. As shown in Figure S7 (Supporting Information), even after 6000 cycles, the current density response remains rapid, and the peak deposition current exhibits minimal attenuation. This sustained electrochemical activity strongly demonstrates that residual trace copper particles provide active sites for subsequent copper deposition, effectively reduces energy barrier for subsequent metal deposition and enhances the deposition rate.

The infrared emissivity during cycling of the device is shown in Figure 2e. The initial cycle exhibited an emissivity modulation range of Δε = 0.43. After 3000 cycles, the device maintained nearly identical emissivity modulation compared to the initial cycle, demonstrating negligible performance degradation and being consistent with the results in Figure 2c,d. Following 6000 cycles, the device achieved emissivity switching from 0.73 in the high‐emissivity state to 0.40 in the low‐emissivity state, representing 23% attenuation in emissivity modulation capability (Δε = 0.33), demonstrates superior cycle performance compared to Pt‐electrode devices, (Figure S8, Supporting Information, 54% attenuation in emissivity modulation capability after 5000 cycles). Figure S9 (Supporting Information) shows the surface morphology of Ir electrode in the copper reversible electrodeposition device after 8000 cycles, local electrode detachment appeared possibly due to cycling‐induced stress corrosion cracking.^[^ ^36^ ^]^ Compared to traditional Pt electrode, there were fewer residual particles on the surface, indicating weakened stress corrosion effect on Ir electrode surface.

To further evaluate the cycling performance of the Cu‐based RMED, we have compared the cycling performances of dual‐band compatible Cu electrodeposition device based on ultra‐thin Ir electrode on BaF_2_ substrate (3 × 3cm^2^) with other RMEDs reported to date (Figure 2f).^[^ ^9^, ^20^, ^21^, ^23^, ^24^, ^25^, ^26^ ^]^ Comparative studies confirm that the cycling performance of devices prepared by other optimization approaches cannot rival this methodology, underscoring the critical role of electrode engineering in ensuring reliable operation stability. The advantages of Ir electrodes demonstrated in this work originate from the following aspects: 1) excellent electrochemical stability of Ir electrode effectively suppresses electrode dissolution/passivation and maintains structural integrity during cycling, 2) nanostructured Ir film with excellent structural contact and strong adhesion to the substrate maintains low interfacial resistance, minimizes polarization losses, ensures efficient charge transfer at the electrode‐electrolyte interface, and enables uniform metal electrodeposition.^[^ ^21^ ^]^ These combined properties collectively demonstrate Ir’s suitability as an electrode material for long‐term operation stability in copper reversible electrodeposition devices.

### 2.3 Cycling Performances of Large‐Area Rigid Device with Ir‐Based Composite Electrode

To facilitate the transition from laboratory prototypes to manufacturable devices, we developed Ir‐based composite electrode scalable solutions. The inherent resistance of ultra‐thin metal electrodes induces significant ohmic voltage drops between edges and center regions, particularly in large‐scale devices. This effect can severely compromise deposition uniformity or even prevent deposition entirely. Non‐uniform deposition on electrodes may induce dendrite formation or device short‐circuiting, deteriorating cycling performance and even causing device failure. Therefore, an ideal electrode should enable uniform metal film nucleation and growth within the potential window.^[^ ^37^ ^]^ To address the issue, we developed an innovative Ir/Au grid composite electrode (optical image and schematic, Figures S10 and S11, Supporting Information). Au was chosen for the grid due to its electrochemical inertness and resistance to dissolution at operational voltages. The electrical conductivity of grid composite electrode markedly exceeds that of nanoscale metallic thin films, as evidenced by the sheet resistance values of 171 Ω sq^−1^ for 4 nm Ir electrodes versus 108 mΩ sq^−1^ for 200 nm Au counterparts, demonstrating a three‐order‐of‐magnitude decrease. The minimal ohmic losses in the Ir/Au grid electrode ensure that all four edges of each 1.44 cm^2^ segmented zone remain equipotential with the device's external boundaries, enabling 4 nm Ir layer to uniformly modulate emissivity within each zone. This design strategically sacrifices minimal infrared transmittance to significantly improve conductivity.

**Figure 3:**
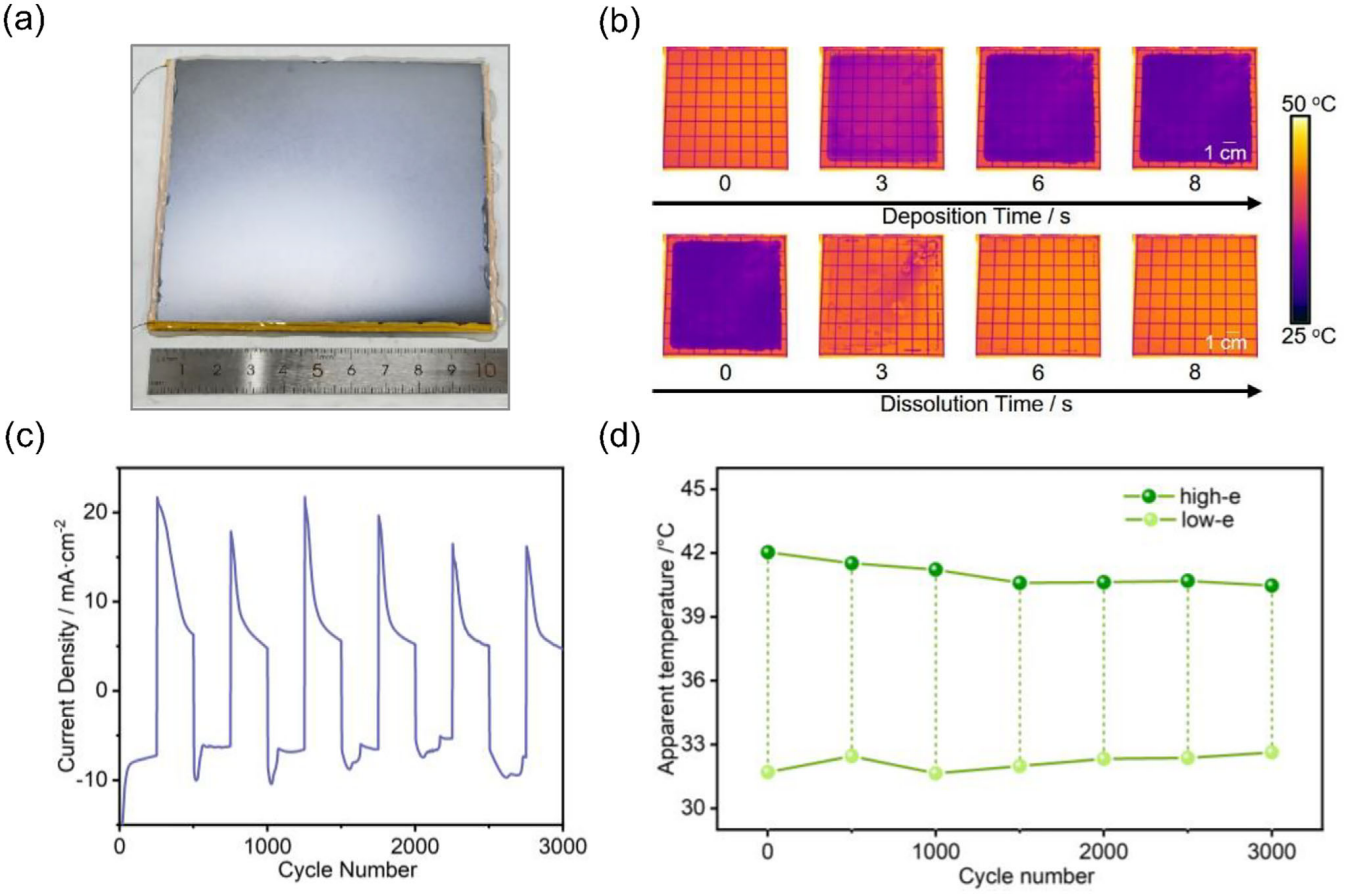
Cycling performances of large‐area reversible copper electrodeposition device with Ir/Au composite grid electrode deposited on Si substrate (10 × 10 cm^2^). a) Optical image of the fabricated device. b) Real‐time IR images of the device during deposition/dissolution. c) Current density profiles during device cycling. (d) Radiative temperature variation of the device during cycling.

**Figure** **3**a presents an optical photograph of the fabricated large‐scale rigid device (10 × 10 cm^2^) incorporating Ir/Au grid composite electrode (on Si substrate). Figure 3b illustrates the deposition and dissolution processes of the large‐area device through infrared thermal imaging, revealing the device's state at different times. Furthermore, the Ir/Au grid composite electrode is clearly visible, and the metal deposition and dissolution are highly uniform across entire device. Besides, the composite electrode demonstrates superior performance characteristics: enhanced conductivity permits the scaled‐up device to achieve 8 s response time comparable to small‐area devices while sustaining sequential metal deposition from periphery to center. Remarkably, the dissolution process completes in just 4 s, fully restoring the initial high emissivity state due to accelerated self‐dissolution of the Cu layer by high uniformity of electric field. The device's deposition current density rapidly decreases from an initial 17.4 to ≈7.4 mA·cm^−2^ within 2 s, representing tenfold faster than conventional small‐area devices (3 × 3 cm^2^). This behavior indicates the fast conversion from nucleation‐limited to diffusion‐controlled regime. The chronoamperometric curves of small‐scale and large‐scale devices (Figure S12, Supporting Information) also show that composite grid electrodes significantly improve the performance and response speed of large‐area devices. During deposition, the peak current density increased severalfold, response time shortened from 20 to 8 s, and deposition current density decayed rapidly. This enhancement originates from the composite electrode’s uniform electric field—enabled by suppressing local current fluctuations and metal ion concentration gradients—along with accelerated reversible Cu deposition/stripping kinetics.

To evaluate the cycling performances, we conducted cyclic testing (±2.5 V, 8 s switching, 16 s/cycle), the devices maintain 70% of the initial current density after 3000 cycles (Figure 3c), indicating small and uniform internal resistance within whole device. This feature is conducive to uniform and reversible electrodeposition. Specifically, the device maintained stable infrared emissivity modulation throughout 3000 cycles and exhibited surface temperature variation of 9.5 °C during the initial cycle and maintained 7.9 °C after 3000 cycles, only decreased by 16%, demonstrating robust dynamic infrared radiation modulation (Figure 3d; Figure S13, Supporting Information). After 3000 cycles, irreversible metal agglomeration was observed on the electrode, resulting in partial loss of reversible electrodeposition area due to inefficient charge transfer. Due to the use of copper tape to enhance in‐plane electrode conductivity, metallic grid near copper tape is prone to detachment caused by the Joule heating effect. This degradation mechanism is corroborated by chronoamperometry (Figure 3c), showing progressive current density reduction that mirrors conductivity losses owing to grid detachment. Real‐time monitoring through infrared thermal imaging and radiation temperature measurements confirmed the devices with composite electrode have exceptional cycling stability and spatial deposition uniformity. The successful transition of concept demonstration to large‐area devices constitutes a pivotal advancement toward deployable reversible electrodeposition‐based emissivity control systems.

### 2.4 Cycling Performances of Flexible Device with Ir‐Based Composite Electrode

**Figure 4:**
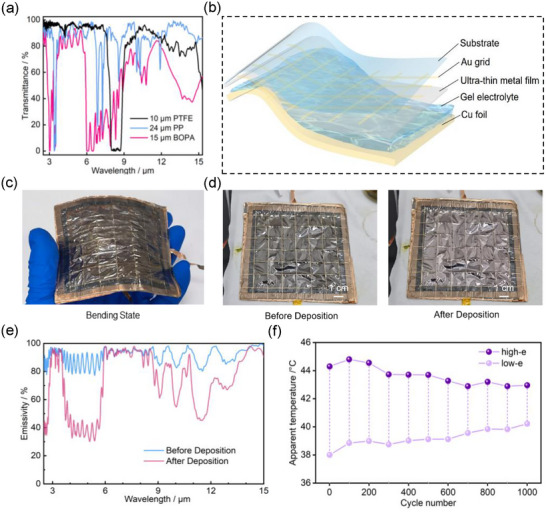
Cycling performances of flexible reversible copper electrodeposition device. a) Transmittance curves of different polymer flexible substrates. b) Schematic diagram of large‐area (8 × 8 cm^2^) reversible copper electrodeposition device with composite grid electrode on flexible substrate. c) Large‐area (8 × 8 cm^2^) reversible copper electrodeposition device Ir/Au grid composite electrode on BOPA substrate demonstration under bending deformation. d) Large‐area (8 × 8 cm^2^) reversible copper electrodeposition device Ir/Au grid composite electrode on BOPA substrate characterization pre‐ and post‐deposition. e) IR emissivity changes of large‐area (8 × 8 cm^2^) reversible copper electrodeposition device Ir/Au grid composite electrode on BOPA substrate. f) Radiative temperature variation of large‐area (8 × 8 cm^2^) reversible copper electrodeposition device Ir/Au grid composite electrode on BOPA substrate during cycling.

To advance the practical application of these devices, we explored strategies to transform rigid devices into flexible configurations. To assess suitability of flexible substrate alternatives for device fabrication, we systematically compared the optical properties of flexible substrates, including polypropylene (PP) film, polytetrafluoroethylene (PTFE) film, and biaxially oriented polyamide (BOPA) film. **Figure** **4**a shows the infrared transmittance of these bare substrates in the 3–14 µm wavelength range: ≈85.7% for PP film, 80.8% for PTFE film, and 55.3% for BOPA film. Although the total transmittance of BOPA film is relatively low, its transmittance in 3–5 µm and 8–14 µm wavelengths can reach 66.5% and 61.2%, respectively. Post deposition characterization of all Ir‐coated substrates revealed <10% reflectance, with significant conversion of transmittance to absorption, demonstrating infrared modulation potential.

In addition, mechanical compatibility issues emerged during fabrication. PP showed poor thermal stability with visible damage after Ir deposition, PTFE's surface inertness and ultra‐flexibility led to coating difficulty and high sheet resistance (4.1 kΩ sq^−1^). In contrast, BOPA film exhibits excellent thermal stability and mechanical toughness, maintaining structural integrity without deformation during the metal electrode evaporation process. Furthermore, corona treatment significantly enhanced its adhesion to metallic coatings by improving surface wettability and promoting interfacial bonding. 4 nm Ir layer deposited via evaporation on BOPA film achieves sheet resistance of 133 Ω sq^−1^, which is slightly lower than that of 4 nm Ir on Si rigid substrate. These results suggest that BOPA substrate presents competitive alternative to conventional rigid substrate, offering comparable electrical performances while retaining good flexibility.

Based on preliminary screening, we fabricated 3 × 3 cm^2^ flexible devices using 4 nm Ir electrode on BOPA flexible substrate, achieving emissivity modulation of Δε = 0.31 (3–5 µm) and 0.20 (8–14 µm) under −2.5 V bias. However, the current density is lower compared to rigid devices, possibly due to hindered ion transport and suppressed Cu deposition through the incorporated porous filter membrane in the electrolyte layer. To optimize performance and scale up, we designed 8 × 8 cm^2^ flexible device through incorporating ultra‐thin Ir/Au grid composite electrode. The flexible device, exhibiting a sandwich architecture with Ir‐Au composite working electrode on flexible substrate, copper foil counter electrode, and intervening gel electrolyte (Figure 4b), maintains excellent mechanical stability under bending (Figure 4c).

The devices deliver uniform metal deposition/dissolution with high active‐area utilization despite minor wrinkles and bubbles inherent to flexible substrates. Uniform color transition from brown to reflective copper within 8 s deposition time (Figure 4d) demonstrates successful integration of ultra‐thin Ir/Au mesh electrodes with flexible substrates for large‐area adaptable devices. This architecture delivers reduced sheet resistance and increased current density, thus enabling faster modulation (8 s transition time). Furthermore, the device exhibits excellent emissivity modulation (Δε = 0.32 in 3–5 µm and 0.18 in 8–14 µm) (Figure 4e).

Figure S14 (Supporting Information) demonstrates relatively uniform apparent radiant temperature distribution across the BOPA‐film‐based device, though minor wrinkles inevitably appear without affecting metal deposition/dissolution in corresponding regions. Thermal imaging reveals temperature inconsistencies in certain areas, attributable to inherent wrinkling in flexible devices that prevents the flatness achievable in rigid configurations. The chronoamperometric curves (Figure S12, Supporting Information) and infrared thermal images (Figure S15, Supporting Information) indicate that composite grid electrode can also effectively suppress ohmic potential drop and enhance metal dissolution rate for flexible devices. After 500 cycles, localized copper residue becomes visible, causing diminished temperature variation. This effect exacerbated after 1000 cycles due to grid delamination and consequent conductivity degradation in these regions(Figure S16, Supporting Information). Notably, the lower‐left grid area exhibits significant temperature rise (reaching 66 °C) upon voltage application, indicating substantial Joule heating that accelerates grid electrode detachment. After 1000 cycles, extensive left‐side grid loss reduces conductivity, increasing ohmic potential drop and diminishing overall device response. Ion transport between two electrodes is partially impeded, exerting profound impact on the stability of deposition and dissolution processes within device. Chronoamperometric curve for cycling process of flexible large‐area device (Figure S18, Supporting Information) reveals that the decay rate of large‐area flexible devices is significantly faster than that of large‐area rigid devices (Figure 3c). A key reason is the inferior adhesion between the grid metal electrode and the flexible substrate compared to its rigid counterpart. Under identical cycling conditions, grid electrode on flexible device undergoes premature detachment, which significantly undermines the cycling performance of device. Consequently, attenuation of infrared radiation temperature variation in flexible devices during cycling is more rapid than that in rigid devices. Post‐1000 cycling tests confirmed retained radiation temperature control (Figure 4f), though progressive performance degradation was observed. The preserved Ir/Au grid electrode contact ensures uniform emissivity modulation even with cyclically reduced current density, where prolonged operation compensates for efficiency losses to sustain consistent modulation amplitudes during extended testing. The experimental results collectively demonstrate both the viability of scalable flexible devices and the critical requirement for improved grid longevity.

The accelerated performance decay in flexible devices under cycling compared with the rigid ones possibly stems from the mechanical instability and electrochemical instability. Specifically, the mechanical instability originates from the interfacial contact failure during metal deposition‐dissolution cycling. First, during the electrodeposition of Cu films, isolated grains coalesce to minimize surface energy, generating significant tensile stress. This stress due to the Volmer‐Weber growth mode,^[^ ^20^ ^]^ intensifies with higher deposition rates and smaller grain sizes. The underlying mechanism involves the chemical potential gradient between surface adatoms and grain boundaries, which drives adatom insertion into the boundaries. The accumulated stress in deposited metal film results in severe, widespread fractures and delamination due to stress corrosion cracking.^[^ ^36^ ^]^ Furthermore, during the deposition and dissolution of metal films, interfacial contact loss at the electrode‐electrolyte interface often occurs, a process primarily manifested by the formation of nanoscale voids. These voids significantly increase the interfacial impedance and provide favorable sites for metal filament growth during deposition.^[^ ^38^ ^]^ This, in turn, raises the risk of internal short circuits and ultimately severely compromises the device's performance and cycling stability. The electrochemical instability includes interfacial side reactions and ion transport limitations (including concentration polarization). On the one hand, in flexible devices, compromised interface stability promotes electrolyte decomposition at the electrode surface (e.g., forming a solid electrolyte interphase or undesired gases). This irreversible side reaction consumes active metal ions and electrolyte, thereby accelerating capacity fade and increasing internal resistance. One the other hand, the presence of microcracks and interfacial gaps caused by mechanical stress severely hinders ion transport, leading to pronounced concentration polarization.^[^ ^39^ ^]^ This effect narrows the emissivity modulation range and ultimately accelerates cycling performance degradation. These findings underscore that, beyond electrode optimization, enhancing the mechanical integrity and interfacial adhesion is paramount for developing next‐generation robust flexible devices. Future work will focus on employing selfhealing polymers or stress‐relief structures to mitigate these issues for boosting cycling stability.

### 2.5 Generality of Ir Electrode for Multispectral‐Compatible Device

**Figure 5:**
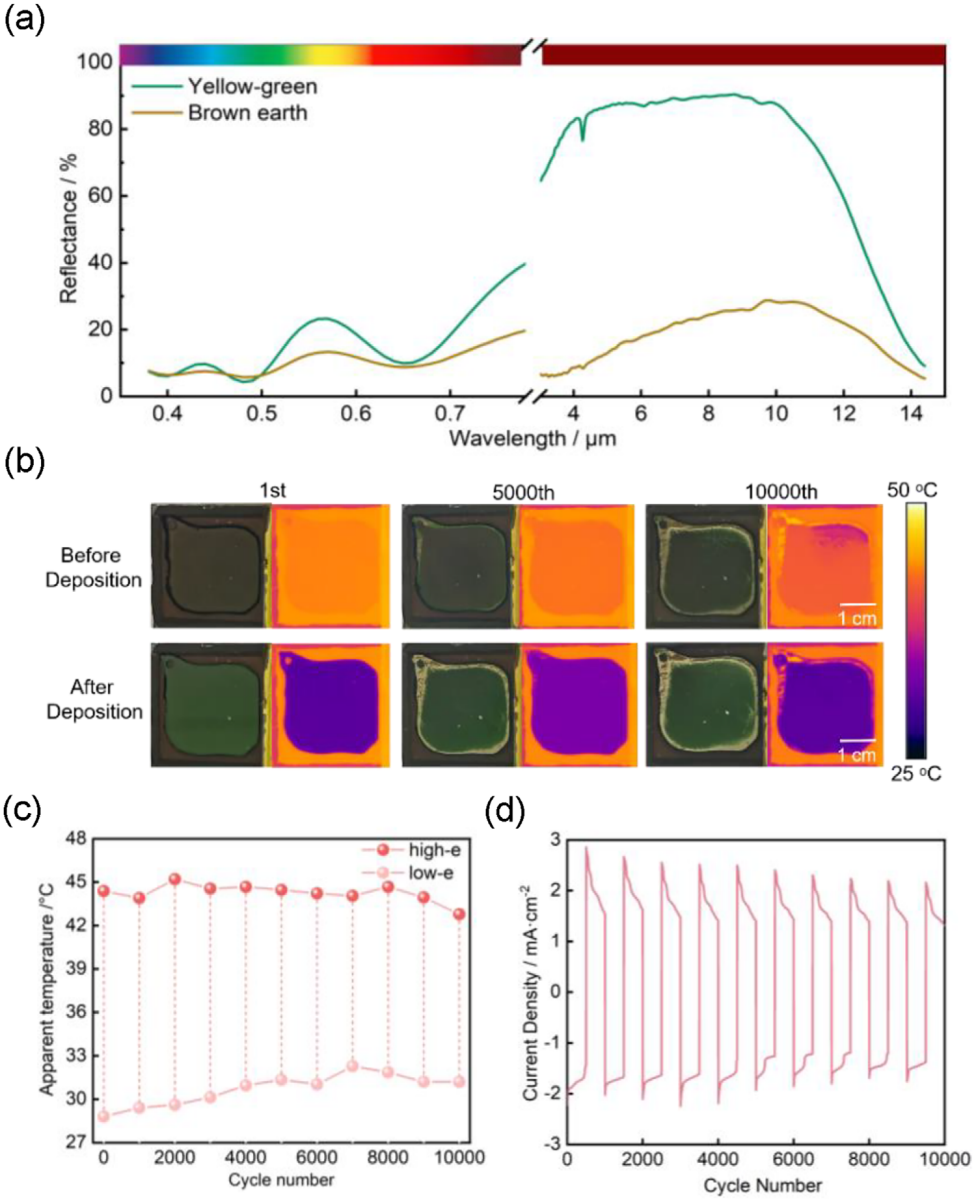
Cycling performances of dual‐band compatible copper‐based device ultra‐thin Ir electrode and Cr_2_O_3_ interlayer and on BaF_2_ substrate (3 × 3 cm^2^). a) Reflectance spectra of the device across visible and infrared bands. b) Optical images and IR images of the device during cycling. c) Radiative temperature evolution of the device during cycling. d) Current density‐ cycling number relationship of dual‐band compatible copper‐based device.

To demonstrate the generality of our electrode engineering, we redesigned the device architecture through strategic optical and electrical optimization to realize the visible‐IR dual‐band compatible devices. We introduced Cr_2_O_3_ optical interference layer between BaF_2_ substrate (dual‐band transparency in visible and infrared wavelengths, BaF_2_ substrate IR transmittance(0.94 in 3–5 µm, 0.87 in 8–14 µm), Figure S1; Table S2, Supporting Information) and Ir electrode to enable simultaneous visible color modulation through constructive interference effects and infrared emissivity control through reversible Cu electrodeposition. The device (3 × 3 cm^2^) exhibited dual‐band dynamic modulation performance (**Figure** **5**a,b): 1) in the visible range (490–590 nm), reflectance changes induced distinct color transition from brown‐yellow to green, 2) in the infrared range (3–14 µm), reflectance increased from 19.1% to 74.8% (Δε = 0.56). This architecture enables independent, dynamic modulation from visible to IR, meeting modern multispectral camouflage requirements.

The dynamic modulation mechanism is the synergistic interaction between reversible metal electrodeposition and the Cr_2_O_3_ interference layer (Figure S17, Supporting Information). Initially, the Cr_2_O_3_ layer of specific thickness displays a characteristic color via interference. Upon Cu electrodeposition on the underlying Ir film, its high reflectivity boosts the Cr_2_O_3_ lower‐interface reflection intensity, driving visible dynamic color changes. A key enabler for simultaneous compatible control is Cr_2_O_3_’s dual properties: low visible absorption, high visible interference intensity, and infrared transparency. Visible color changes rely on the deposited metal‐interference layer synergy, while infrared emissivity modulation depends solely on metal deposition extent. These independent effects enable dual, separate control over visible color and emissivity modulation.

IR thermal imaging and electrochemical cycling tests revealed exceptional device stability. Figure 5b–d shows consistent performance after 10 000 cycles. Specially, consistently large and spatially uniform apparent radiation temperature modulation is sustained over 10 000 operational cycles; which undergo ≈25% decay from ΔT = 15.6 °C of initial cycle to ΔT = 11.6 °C. The minimal current density variation, which maintained 75% of the initial value after 10000 cycles, confirms the outstanding electrochemical durability of the Ir electrode.

As shown in Figure 5d, a rapid transient spike in current density quickly decays to a steady state, being consistent with the behavior observed in Figure 3c and Figure S18 (Supporting Information). This transient spike primarily originates from the coupled kinetics of non‐faradaic processes (electric double‐layer charging) and faradaic processes (redox reactions) during the voltage step transition. The underlying mechanism is elaborated as follows. The nature of the phenomenon may be attributed to the electric double‐layer charging current, also known as solid electrolyte interface (SEI). When a voltage is applied or switched across the device, an intense electric double‐layer charging/discharging process occurs. This non‐faradaic process generates a substantial transient current, manifesting as a rapid current spike. Specifically, localized fracture and rapid self‐repair of the SEI on the electrode surface can induce transient current fluctuations. Furthermore, during cycling, voids formed by metal deposition/dissolution or SEI reconstruction may generate tiny conductive dendrites. When these dendrites cause a short circuit, the resulting high current rapidly burns them out, allowing the device to recover—a process that manifests as a sharp current pulse.^[^ ^40^ ^]^ Analogous to an “inrush current”, this phenomenon is characterized by an instantaneous response that cannot be sustained; it rapidly decays once the electric double‐layer becomes fully charged. The reason for its rapid dissipation may be closely related with the resistor‐capacitance (RC) time constant. The entire device, particularly the flexible device with multilayer structure, can be equivalently modeled as an RC circuit. The rapid dissipation of the current spike corresponds to a small RC time constant in this equivalent circuit. This indicates that ions can rapidly undergo initial rearrangement at the electrode‐electrolyte interface under the applied electric field, thereby quickly establishing electrochemical potential equilibrium. Overall, the observed current spike is a characteristic feature of the device transitioning from a transient to a steady‐state response under voltage excitation, demonstrating the relatively fast initial ion response speed of our device.

Progressive color evolution from initial yellow‐green to dark green is observed with cycling, accompanied by edge‐localized golden crack‐patterned deposits that impair optical uniformity after 10 000 cycles (Figure S19, Supporting Information). The repeated Cu deposition‐dissolution processes generate escalating internal stresses, culminating in stress‐corrosion‐induced cracking and leading to interfacial delamination. Whereas the device maintained functional visible‐infrared modulation capability throughout testing. Only minor peripheral deposition failure occurred, with over 85% of the active area maintaining full performance, thus confirming reliable operational stability. Consequently, this ultra‐thin metal electrode design exhibits exceptional resistance to current‐induced stress corrosion during cycling and demonstrates excellent generality for multispectral compatible devices.

## 3 Conclusion

This study presents a transformative electrode optimization strategy that fundamentally enhances cycling performances of reversible metal electrodeposition device. Designing ultrathin Ir metal electrodes achieves unprecedented device cycling stability due to inherent chemical inertness, superior conductivity, and mechanical robustness. This electrode engineering approach effectively suppresses stress‐induced cracking, electrolyte corrosion, and electrochemical oxidation during cycling operation. To demonstrate the feasibility and generality of Ir electrode for practical devices, we designed Ir‐Au grid composite electrode for large‐area devices, which ensures uniform conductivity and preventing island‐like deposition‐induced failure, the 10 × 10 cm^2^ rigid Si‐based device exceeds 3000 cycles, while the 8 × 8 cm^2^ bend‐tolerant BOPA‐based flexible device sustains >1000 cycles. Integrating Cr_2_O_3_ optical interference layer with ultrathin Ir electrodes produces the dual‐band modulation device (BaF_2_ substrate, 3 × 3 cm^2^), simultaneously controlling visible‐light coloration and mid‐far‐infrared emissivity (Δε = 0.56) and sustaining operational longevity over 10 000 cycles. This study highlights the critical role of electrode design in determining the cycling performance of RMED, which demonstrates that an optimized electrode architecture significantly improves cycling durability, thereby facilitating the development of stable and scalable devices suited for practical applications.

## 4 Experimental Section

### Electrode Preparation

Square silicon wafers (Zhejiang Lijing Silicon Materials Inc.) and BaF_2_ substrates (Shanghai Purui Duo Optical Materials Co., Ltd.) were used as the rigid working electrode substrate. PP film (Chemplex Industries Inc.), PTFE film (Hongfu Insulating Materials Factory) and BOPA film (Cangzhou Mingzhu Diaphragm Technology Co., Ltd.) were used as the flexible working electrode substrate. The thin metal films were directly evaporated onto these substrates using an electron‐beam evaporation system (Kurt J. Lesker PVD 75), the operating parameters are listed in Table S4 (Supporting Information). The ultra‐thin Ir/Au mesh composite electrode was used for fabrication of large‐area devices. With the aid of a custom‐made stainless steel mask, Au strips with width of 400 µm and thickness of 200 nm were deposited at intervals of 12 mm on an infrared‐transparent substrate, forming Au mesh by sequentially depositing in both horizontal and vertical directions. Subsequently, the mask was removed, and a 4 nm thick Ir layer was deposited to create the ultra‐thin Ir/Au mesh composite working electrode. Two types of counter electrodes were prepared: for the silver reversible electrodeposition device, a glass substrate was used, and a 15 nm Pt layer followed by a 200 nm Ag layer were continuously evaporated following the same procedure as the working electrode, detailed production process diagram provided by Figure S20 (Supporting Information). For the copper reversible electrodeposition device, a commercially available copper foil (from Kunshan Shengshijingshin New Material Co., Ltd.) was used as the counter electrode.

### Electrolyte Preparation

The electrolyte for the silver reversible electrodeposition system comprises 0.5 M AgBr (Aladdin), 0.5 M TBABr (Aladdin), and 10 wt.% PVB (Sinopharm Chemical Reagent) dissolved in DMSO (Aladdin). The electrolyte without Br^−^ comprises 50 mM AgNO_3_ (Sigma–Aldrich), 250 mM LiClO_4_ (Aladdin), and 10 wt% PVB dissolved in DMSO. The electrolyte for the copper reversible electrodeposition system comprises 80 mM CuCl_2_ (Aladdin), 0.25 M LiClO_4_, 0.6 mM KI and 10 wt% PVB dissolved in DMSO. All chemicals were mixed and stirred on a hot plate set to 60 °C and 900 rpm for at least 12 h, until the solid was completely dissolved. The prepared solution should be tightly sealed to prevent contact with air, which could lead to the formation of flocculent matter.

### Device Assembly

First, conductive silver paste (SPI) was applied to the side and front of the working electrode, and a silver fiber wire was attached to one side as the electrode lead. After thorough drying, PI tape was applied to prevent the electrolyte from coming into contact with the surrounding silver paste during device assembly. Next, conductive silver paste was used to attach a silver fiber wire to one side of the counter electrode as the electrode lead, and after thorough drying, it was set aside for later use. A Teflon double‐sided tape with a width of ≈4 mm was applied around the counter electrode, leaving a 2 mm wide electrolyte injection port. A dispensing machine was used to apply transparent silicone sealant over the tape on the counter electrode, and the working electrode was then placed on top. After the sealant solidified, additional sealant was applied around the edges of the device for reinforcement. Finally, the electrolyte solution was injected through the reserved port, and sealant was applied to complete the sealing.

### Characterization

The surface morphology of the working electrode was observed using a Sigma 300 scanning electron microscope from Zeiss. Compositional analysis of the ultra‐thin metal electrode and the electrodeposited metal layer was performed using a Smart EDX energy dispersive X‐ray spectrometer from Zeiss. Film thickness was measured using a DEKTAK XT profilometer from Bruker. Infrared reflectance, transmittance, and emissivity of the samples were characterized using a Vertex 70 Fourier transform infrared spectrometer from Bruker. The measurements were conducted in integrating sphere mode, with a sample port diameter of 2.1 cm, a measurement range of 400–4000 cm^−1^, and an incident light angle of 12°. Visible light spectral curves of the devices were measured using a Lambda spectrophotometer from PerkinElmer, with a measurement range of 0.25–0.8 µm, a spectral resolution of less than 0.05 nm in the UV‐vis range, and a wavelength accuracy of ±0.08 nm. Apparent radiation temperature changes of the devices were recorded using a T1050 SC infrared thermal imager from FLIR. The sheet resistance of various thin films was measured with a four‐probe resistivity measurement system (RTS‐9). The electrochemical performance of the devices, including chronoamperometry (CA) and cyclic voltammetry (CV), was tested using a PARSTAT 4000 electrochemical workstation from AMETEK.

### Testing the Effect of Br^−^ on Cycling Performance of Ag‐Based RMED

Including two‐electrode cyclic voltammetry with switching potentials ±2.5 V, linear voltammetry, chronoamperometry, infrared radiation temperature regulation performance of the device under different dissolution times (10 and 15 s) and dissolution voltages (0.8 V), and observation of electrode morphology after cyclic operation in a Br^−^‐free system. Detailed test results are provided in Figure S21 (Supporting Information).

### Dissolution Resistance Tests

Accurate infrared spectroscopic measurements require either inherent open‐circuit stability or application of a protective current to prevent Cu layer dissolution via spontaneous dissolution mechanisms after voltage removal. Dissolution inhibition tests were consequently performed. A comparative study was conducted using three configurations: 30 nm and 1 µm thick Cu electrodes sealed in copper electrolyte, alongside a 30 nm Cu control sample in DMSO. Figure S5 (Supporting Information) shows the dissolution process over 24 h. Detailed test results are provided in Figure S22 (Supporting Information).

### Open‐Circuit Stability Tests

After complete deposition of the device at −2.5 V, open circuit for 6, 24, and 48 h, respectively, and perform infrared reflectance testing. Detailed test results are provided in Figure S23 (Supporting Information).

### Calculation

The absorption (α), reflectance (R), and transmittance (T) of the sample satisfy the following relationship:

(1) $$\begin{array}{c} \alpha +R+T=1 \end{array}$$

By integrating the infrared reflectance and transmittance curves, the average infrared transmittance and average infrared reflectance of the substrate in 3–14 µm wavelength range can be calculated. The average infrared transmittance of the substrate can be expressed as:

(2) $$\begin{array}{c} \;{\bar{T}}_{\left(3-14\cdot \mu m\right)}=\frac{\int_{3\cdot \mu m}^{14\cdot \mu m}T\left(t,\lambda \right)d\lambda }{\int_{3\cdot \mu m}^{14\cdot \mu m}d\lambda }\; \end{array}$$

where ${\bar{T}}_{(3-14\cdot \mu m)}$ is the average infrared transmittance of the sample in 3–14 µm wavelength range. With a wavelength of λ. T(*t,λ*) is the spectral transmittance of the substrate.

Similarly, the average infrared reflectance of the substrate can be expressed as:

(3) $$\begin{array}{c} \;{\bar{R}}_{\left(3-14\cdot \mu m\right)}=\frac{\int_{3\cdot \mu m}^{14\cdot \mu m}R\left(t,\lambda \right)d\lambda }{\int_{3\cdot \mu m}^{14\cdot \mu m}d\lambda }\; \end{array}$$

where ${\bar{R}}_{(3-14\cdot \mu m)}$ is the average infrared reflectance of the sample in 3–14 µm wavelength range. With a wavelength of λ. R(*t,λ*) is the spectral reflectance of the substrate.

According to the blackbody radiation law, emissivity is defined as the ratio of the total radiation emitted by an object to that emitted by a blackbody. This study focuses on the emissivity in 3–14 µm wavelength range, with the reference blackbody radiation temperature set at room temperature (298 K). The calculation formula is as follows:

(4) $$\begin{array}{c} \varepsilon \left(3,14\right)=\frac{\int_{3}^{14}I_{BB}\left(\lambda \right)\varepsilon \left(\lambda \right)d\lambda }{\int_{3}^{14}I_{BB}\left(\lambda \right)d\lambda }\; \end{array}$$

where ε(3, 14) is the emissivity in 3–14 µm wavelength range.$\int_{3}^{14}I_{\mathrm{BB}}(\lambda )\varepsilon (\lambda )d\lambda$ is the total radiation emitted by the device in 3–14 µm wavelength range.$\int_{3}^{14}I_{\mathrm{BB}}(\lambda )d\lambda$ is the total radiation emitted by a blackbody at 298 K in 3–14 µm wavelength range.

According to Kirchhoff’s law, the infrared emissivity (ε) of an object is equal to its infrared absorptivity (α):

(5) $$\begin{array}{c} \varepsilon \left(T,\lambda \right)=\alpha \left(T,\lambda \right) \end{array}$$

By using (Equation 1), the infrared absorptivity of the device, which is equivalent to the spectral curve of the infrared emissivity, can be calculated. Substituting this result into (Equation 4) allows for the determination of the emissivity in 3–14 µm.

## Conflict of Interest

The authors declare no conflict of interest.

## Supporting information



Supporting Information

## Data Availability

The data that support the findings of this study are available in the supplementary material of this article.
