# Supplementary material for: Electrode Engineering for High‐Durability Variable‐Emissivity Devices Based on Reversible Copper Electrodeposition
Source: Adv Sci (Weinh). 2025 Dec 23;13(11):e20497. doi: 10.1002/advs.202520497 (PMC12931165; doi:10.1002/advs.202520497)
Supplement: Supplementary file 1 — Supporting Information [file ADVS-13-e20497-s001.docx]

Supporting Information

**Electrode Engineering for High-Durability Variable-Emissivity Devices Based on Reversible Copper Electrodeposition**

Runyun He^#^, Tianwen Liu^#^, Liqiang Zhang*, Tuoyu Liu, Dan Tang, Wenxia Zhang, Jundong Tao, Tingting Shi, Yijing Song, Haifeng Cheng, Yao Zhang*, and Dongqing Liu*


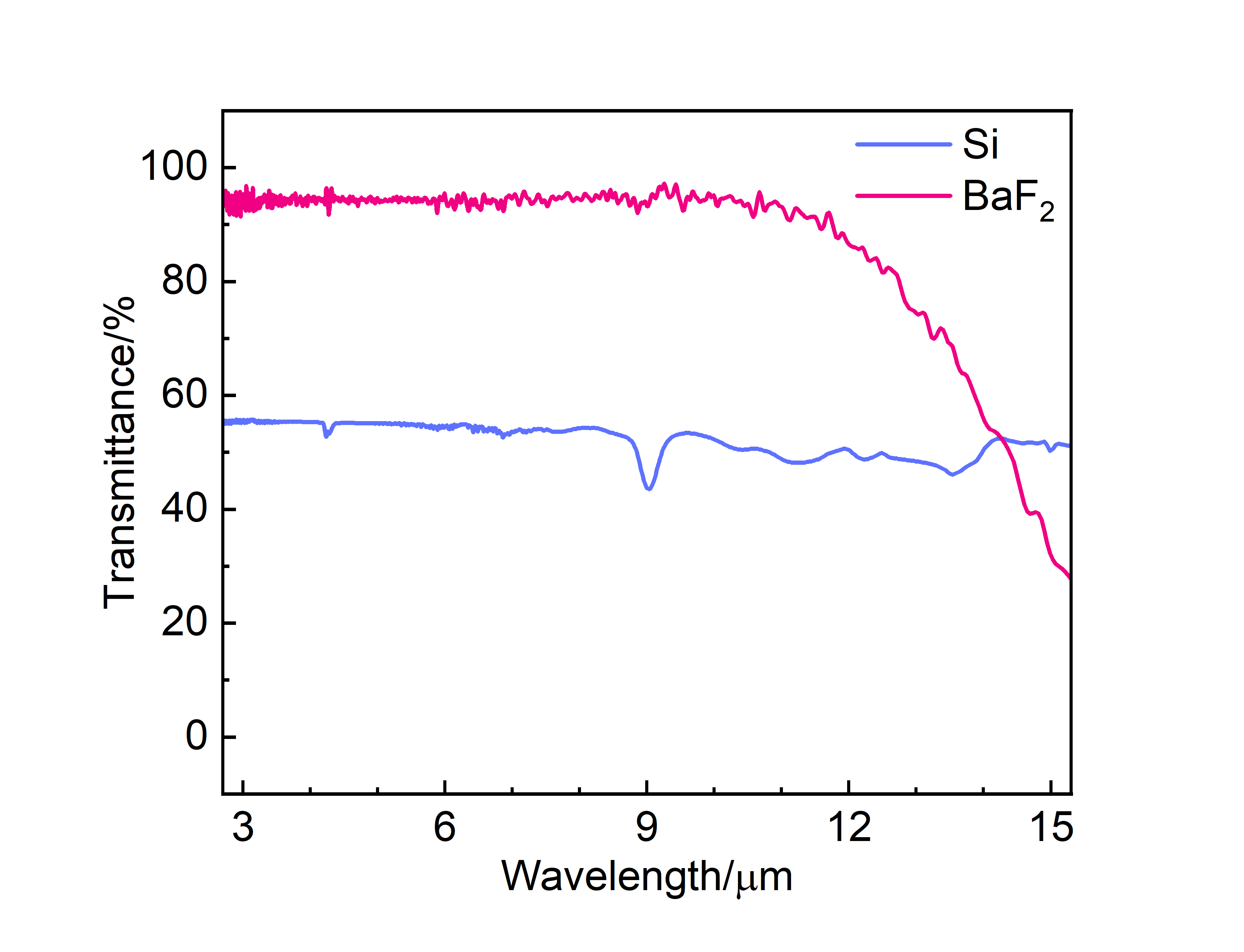


**Figure S1.** Infrared (IR) transmittance curves of Si substrate and BaF_2_ substrate.

Although silicon substrates exhibit low overall transmittance in infrared spectrum, their low cost and suitability for large-scale fabrication make them the preferred substrate for large-area rigid devices. However, since visible-IR dual-band compatible modulation devices require substrates that are infrared-transparent while maintaining visible light transmittance, we must resort to the BaF_2_ substrate. BaF_2_ exhibits exceptionally high infrared transmittance, enabling the fabricated devices to achieve outstanding emissivity control performance in the infrared spectrum.


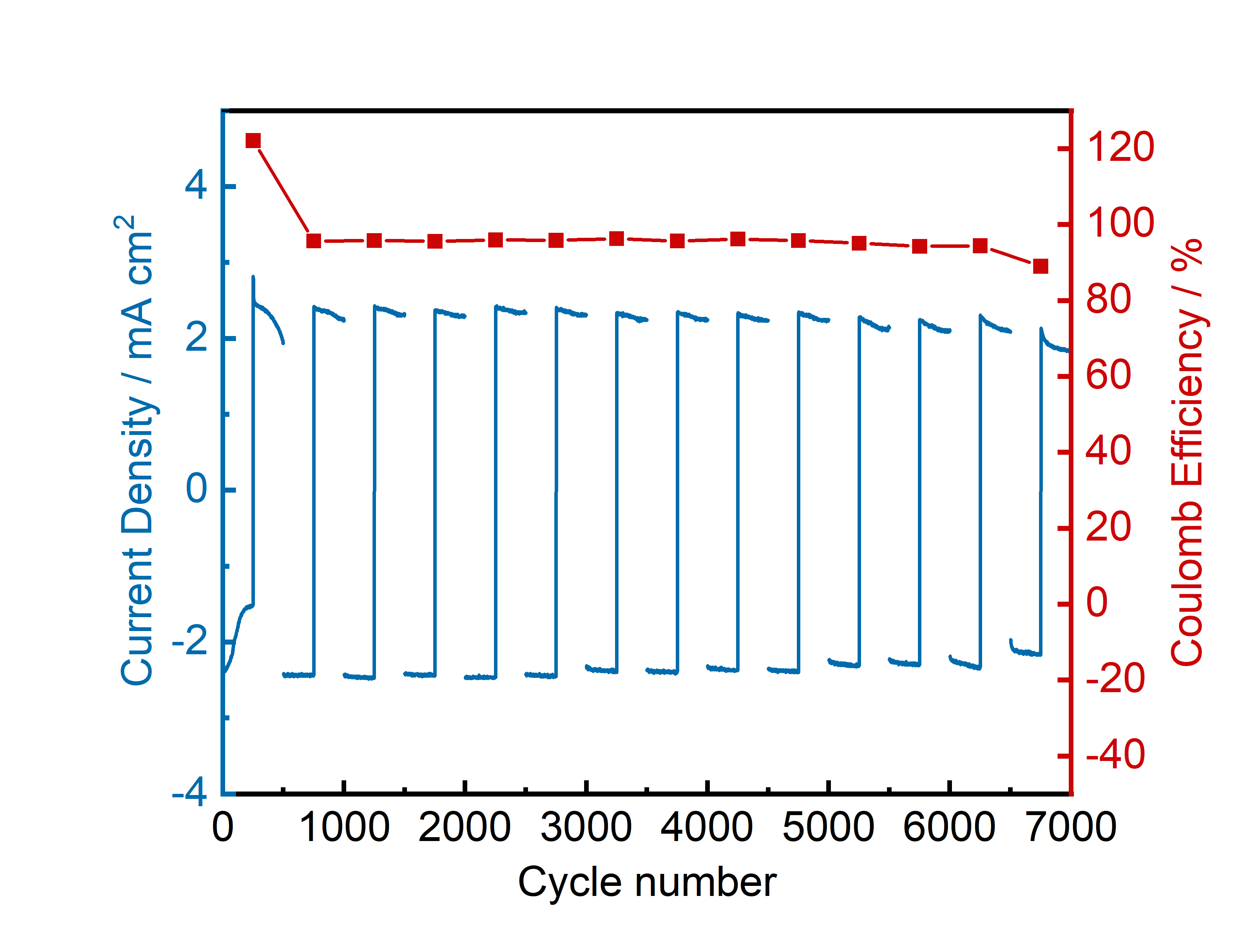


**Figure S2.** Current density curve and coulombic efficiency variation of Pt-electrode-based copper system device during cycling.

**Figure S3.** Scanning electron microscopy (SEM) images of Pt electrode after 7500 electrical cycles (The red circles in the image indicate voids and discontinuities on the electrode surface, while the blue circles mark residual oxide or deactivated/isolated metal particles). These SEM images reveal an uneven surface morphology with dispersed particles, while high-magnification images clearly show isolated residues after 7500 cycles.

Energy dispersive spectroscopy (EDS) analysis of the electrode after copper deposition (Figure S4) revealed that the surface residues consist of oxides and metals (residues marked by the blue circles in the SEM image). The accumulation of oxides originated from the significant potential difference during the platinum electrode’s potential cycling, where the reduction potential of oxides was substantially lower than their formation potential. This difference resulted in a pronounced reduction hysteresis, preventing the complete reduction of all oxides within each cycle.^[1]^ Consequently, unreduced oxides progressively accumulate on the electrode surface during successive cycles. Combined with cycling-induced stress, this leads to a reduction in the active electrode area (discontinuities indicated by the red circles in the SEM image). Combined with four-probe sheet resistance measurements, the sheet resistance in the central region of the cycled electrode (148 Ω sq^-1^) was significantly lower than the initial resistance value (245 Ω sq^-1^), indicating that dead copper residues remain on the electrode surface during cycling. These residual metals can no longer participate in redox reactions and significantly increase the thickness of the working electrode. This leads to an increase in initial reflectance, severely weakening the emissivity modulation effect, and ultimately causing device failure.

**Figure S4.** EDS mappings of (a) copper, (b) platinum, (c) chlorine, (d) oxygen elements and (e) EDS spectrum of 4 nm Pt/Si substrate after Cu electrodeposition.

**Figure S5**. Electrochemical stability of different electrode materials. (a) Linear sweep voltammetry curves; (b) Chronoamperometry curves.


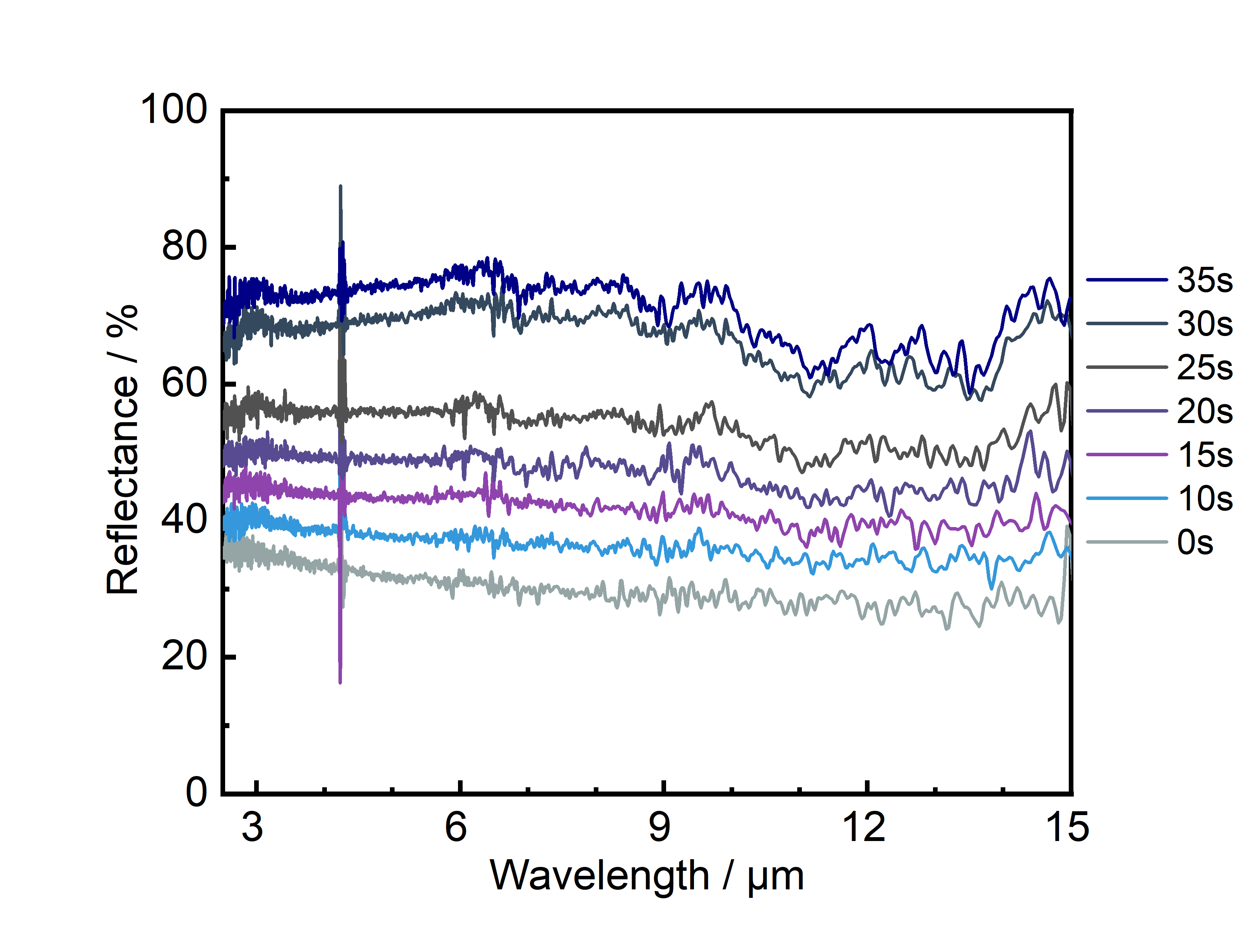


**Figure S6.** IR reflectance changes of copper reversible electrodeposition device with an 4nm Pt electrode.


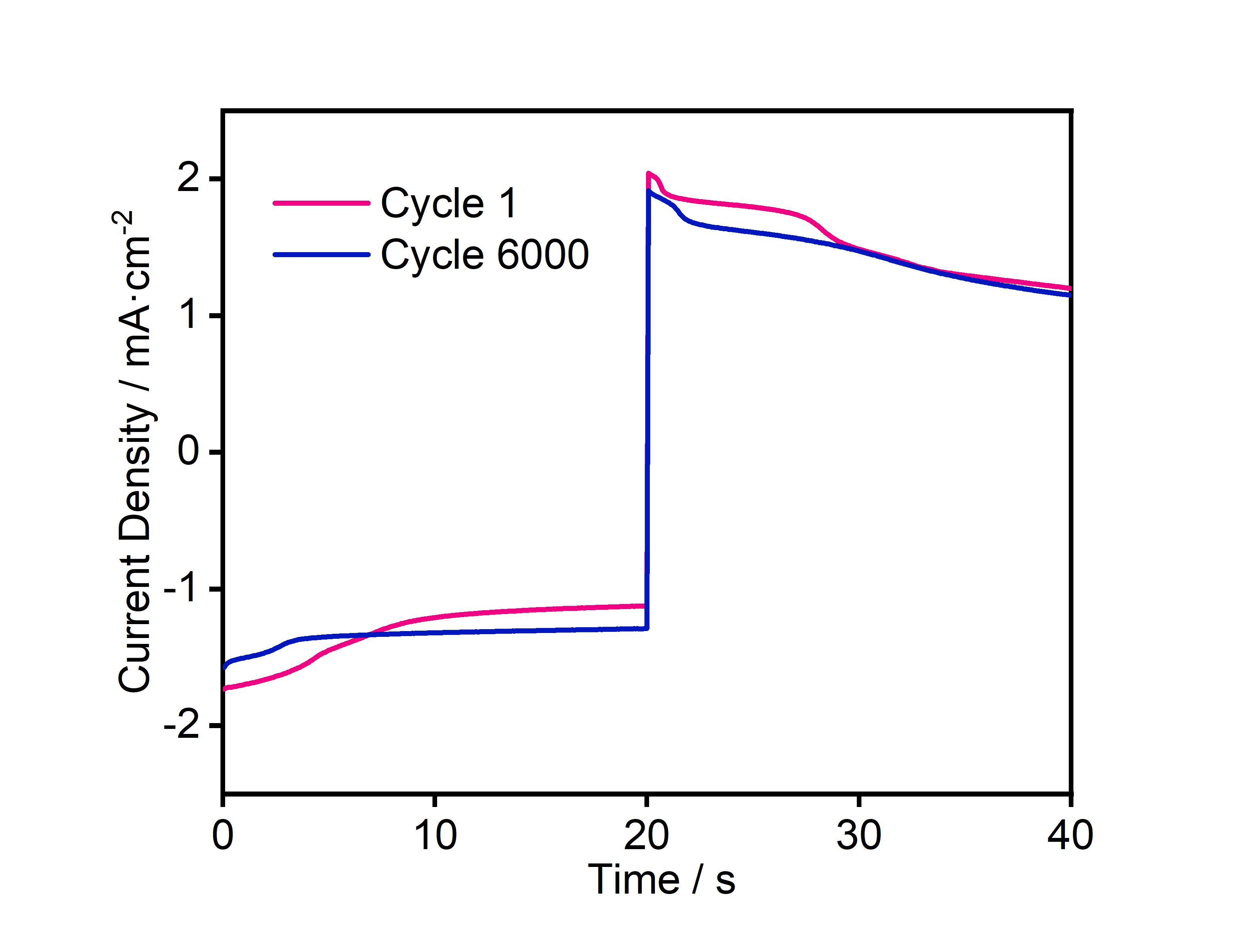


**Figure S7.** Chronoamperometric curves of devices based on Ir electrodes at different cycle numbers.

As observed in chronoamperometric curves, Cu electrodeposition process transitions from nucleation-controlled to diffusion-controlled regime within 10 s. In the initial deposition cycle, current density decreases from 1.7 mA cm^-2^ to approximately 1.2 mA cm^-2^. Over the 6000th cycle, it declines from 1.5 mA cm^-2^ to about 1.3 mA cm^-2^. Notably, even after extensive cycling, the current density response remains rapid, with minimal attenuation of the deposition current peak.

This sustained electrochemical activity strongly indicates that residual trace metal particles function as permanent nucleation sites, providing active centers for copper deposition.^[2]^ This mechanism promotes the concentrated deposition of metal ions on the surfaces of these retained particles, effectively preventing the spatial disorder of nucleation. At the microscopic level, atomic interdiffusion occurs between the residual metal and the subsequently deposited metal. This process optimizes surface energy at the interface, effectively lowering the energy barrier for subsequent metal deposition. Therefore, the highly efficient active sites formed by trace metals deposited on the electrode surface during the initial electrodeposition process are more conducive to achieving subsequent uniform and efficient metal deposition.


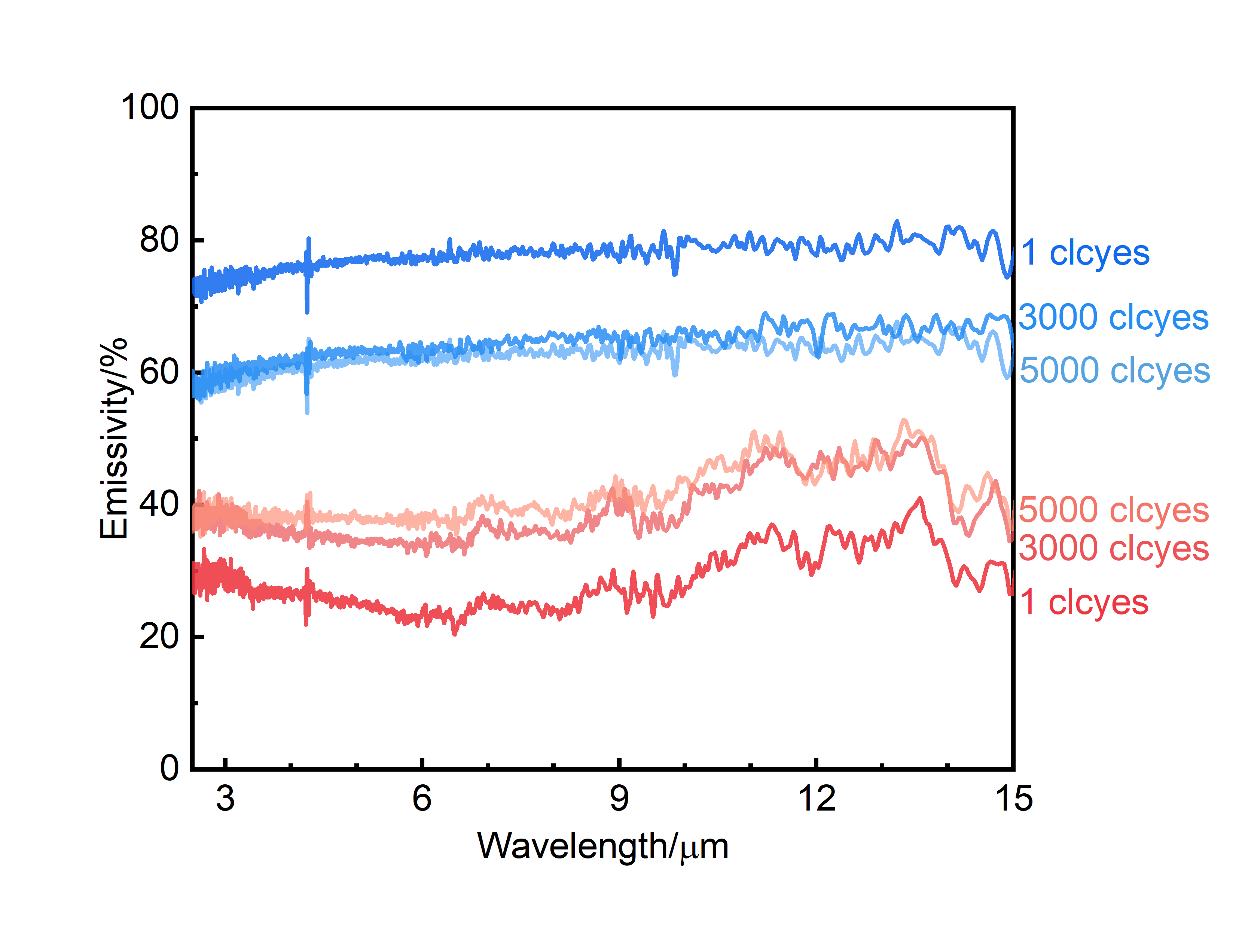


**Figure S8.** IR spectra of Pt-electrode-based copper system device at different cycle numbers.

**Figure S9.** Electrode morphology at (a) 20µm and (b) 1µm of the Ir electrode-based copper reversible electrodeposition device after 8,000 cycles.

**Figure S10.** Image of electrode (with/without Au grid) for large-area device (10 × 10 cm^2^).

**Figure S11.** Schematic of the composite mesh electrode architecture for large-area device (10 × 10 cm^2^).

**Figure S12.** Chronoamperometric curve for (a) rigid large-area device (10 × 10 cm^2^), (b) rigid small-area device (3 × 3 cm^2^), (c) flexible large-area device (8 × 8 cm^2^) and (d) flexible small-area device (3 × 3 cm^2^).

The chronoamperometric curves demonstrate that large-area devices with composite grid electrode exhibit significantly enhanced peak current density during the deposition phase: an approximately 10-fold increase for rigid devices and a 2-fold increase for flexible devices, accompanied by rapid decay in deposition current, indicating notably improved device response speed. This phenomenon can be attributed to the enhanced uniformity of the electric field imparted by composite grid electrode, which reduces local current density fluctuation and concentration gradient and transforms electrodeposition process from an uneven reaction mode to surface-uniform reaction control. This significantly accelerates reversible deposition/stripping kinetics of Cu: ^[3]^ the uniform electric field promotes homogeneous adsorption and nucleation of Cu^2+^ on electrode surface. As deposition proceeds, a dense, smooth, and well-adhered copper layer is formed. During dissolution process, the electric field similarly drives the layer-by-layer and uniform dissolution of copper, thereby preserving structural integrity and electrochemical activity of electrode. In contrast, electrodes without grid electrode suffer from uneven electric field distribution, which tends to cause localized over-deposition and non-uniform dissolution, leading to accumulation of electrochemically inert metallic particles. These residues not only hinder reversible deposition/dissolution process but may also induce localized short circuits, accelerating electrode performance degradation.

**Figure S13.** Real-time IR images of rigid large-area device (10 × 10 cm^2^) during the cycling test.

**Figure S14.** IR spectral response of different flexible substrates. (a) Total IR transmission spectra of 4 nm Ir. (b) Total IR reflection spectra of 4 nm Ir. (c) Total IR absorption spectra of 4 nm Ir.

**Figure S15.** IR images of flexible large-area devices (8 × 8 cm^2^) during deposition and dissolution processes.

This confirms that composite grid electrodes can effectively suppress ohmic potential drop and enhance metal dissolution rates for flexible devices as well, the inherent slight wrinkles of the flexible substrate have no significant impact on electric field distribution or electrochemical kinetics.

**Figure S16.** Real-time IR images of large-area (8 × 8 cm^2^) reversible copper electrodeposition device Ir/Au grid composite electrode on BOPA substrate during the cycling test.

**Figure S17.** Schematic diagram of dynamic emissivity modulation mechanism of the visible-infrared compatible regulation device. ^[4]^

The core of the dynamic modulation mechanism lies in the synergistic interaction between reversible metal electrodeposition and optical interference within the Cr_2_O_3_ layer. As shown in Figure S17, introducing Cr_2_O_3_ layer between the BaF_2_ substrate and the ultrathin metal electrode Ir results in significant changes in the reflection spectrum when the nanometer-thick Cr_2_O_3_ layer acts as a loss medium on the deposited metal surface, thereby inducing color shifts. In the initial state, a Cr_2_O_3_ layer of specific thickness exhibits a corresponding color due to constructive interference effects in the visible light spectrum. As the electrodeposition process progresses, highly reflective Cu gradually deposits onto underlying Ir film, enhancing visible light reflection at the lower interface of the Cr_2_O_3_ layer. This reflection enhancement induced by Cu deposition is crucial for achieving dynamic color changes during the electrodeposition process.^[4]^

The device achieves compatible regulation of visible color and infrared emissivity primarily through three synergistic factors, including interference light intensity of interference layer, infrared transparency of interference layer and the synergistic adaptability between the deposition layer and the interference layer.

First, the Cr_2_O_3_ interference layer features weak absorption in both visible and infrared bands (main absorption concentrated in the ultraviolet region), minimizing energy loss during light propagation. This ensures the interference light retains sufficient intensity to produce a distinct, observable interference phenomenon—laying the foundation for visible modulation.

Second, as an inorganic oxide, the ultra-thin Cr_2_O_3_ layer exhibits exceptional infrared transparency owing to its extremely low infrared absorption rate. Notably, the introduction of this layer barely impairs the device’s dynamic infrared emissivity modulation capability, safeguarding the infrared regulation function while enabling visible interference.

Third, the two modulation mechanisms operate independently yet synergistically: visible spectrum regulation relies on the synergy between structural thickness variations induced by Cu deposition/dissolution and the Cr_2_O_3_ interference effect; in contrast, infrared band modulation is predominantly determined by the extent of metal deposition.

Collectively, these three factors—sufficient interference light intensity, infrared transparency of the interference layer, and the synergistic adaptability between the deposition layer and the interference layer—enable the device to simultaneously achieve visible color changes and infrared emissivity modulation with high compatibility.


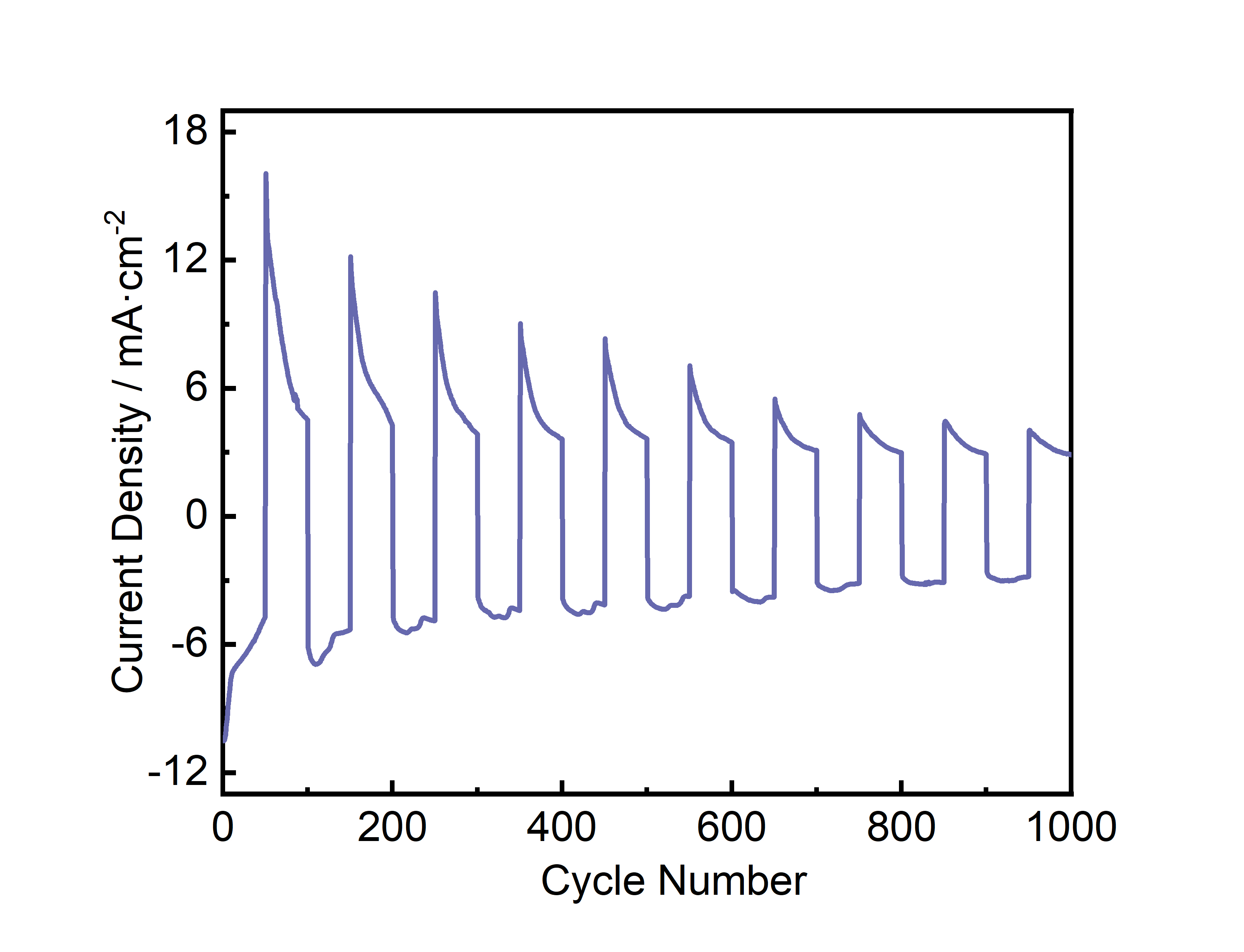


**Figure S18.** Chronoamperometric curve for cycling process of flexible large-area device (8 × 8 cm^2^).

The chronoamperometric curves at different cycle numbers reveal gradual decrease in current density with increasing cycles. Notably, the large-area flexible device exhibits a more rapid decline compared to large-area rigid device (Figure 3c, Manuscript). This finding is corroborated by the increased overall working electrode resistance caused by the detachment of the grid electrode shown in Figure S16 (supporting information), indicating that the adhesion between the flexible substrate and the composite metal mesh electrode remains insufficient. This is a critical factor affecting the cycling lifetime of flexible devices.

**Figure S19.** Visible color and IR image of multi-band compatible control device. (a) Before Deposition; (b) After Deposition.

**Figure S20.** Fabrication process of metal grid-ultrathin metal composite electrodes.

**Figure S21.** Tests exploring halide ions’ impact on cycling performance in Ag-based reversible metal electrodeposition device (RMED) demonstrated that bromide ions inevitably induce detrimental side reaction, whereas the absence of bromide ions compromises the reversibility of silver electrodeposition. (a) Two-electrode cyclic voltammetry curve with a switching potential of ±2.5 V, (b) Linear sweep voltammetry curve, (c) Current density variation. Radiative temperature variation of the device during cycling with dissolution time of (d) 10 s and (e) 15 s, (f) Radiative temperature variation of the device during cycling at dissolution voltage of 0.8 V, (g) IR images of the device after 50 cycles with dissolution time of 10 s and 15 s, (h) Electrode images of the device after cycling failure without Br^-^.

Ag-based RMED devices were selected as a reference system due to the widespread use of Ag in this research field and its established role as a benchmark material. To systematically elucidate the failure mechanisms of Ag-based systems, we have structured analysis into three key aspects:

(1) Electrochemical reactions in Ag-based system: Under negative bias, Ag^+^ ions are reduced and deposited as metallic Ag on the working electrode, while Ag dissolution occurs at the counter electrode to maintain charge balance. The electrolyte, containing AgBr and TBABr, involves two primary redox couples: Ag/Ag^+^ at the working electrode and Br^-^/Br_3_^-^ at the counter electrode. Cyclic voltammetry shows distinct cathodic peaks at -0.5 V (Ag deposition/dissolution) and around -2 V (Br^-^/Br_3_^-^ redox reaction). The latter reaction generates strongly oxidizing Br_3_^-^ species, which diffuse to the working electrode and re-oxidize Ag, significantly degrading the device’s cycling performance.

(2) Influence of dissolution conditions on cycling stability: Experimental results confirm that dissolution time and voltage critically influence side reactions. Extending the dissolution time from 10 s to 15 s enhances Br_3_^-^ formation, reducing the cycle life from 90 to 50 cycles and promoting electrode cracking and conductive island formation. In contrast, lowering dissolution voltage to 0.8 V effectively suppresses side reactions, extending the cycle life to approximately 600 cycles. These results confirm the direct correlation between the Br^-^/Br_3_^-^ redox process and device failure.

(3) Role of bromide ions in reversibility of Ag-based devices: To eliminate bromide-related side reactions, bromide-free electrolyte (AgNO_3_-LiClO_4_) with Ag counter electrode was implemented. Although this system avoids Br_3_^-^ generation, the absence of AgBr complexes severely compromises uniformity of Ag deposition. This leads to prolonged deposition time, increased cycling stress, and rapid device failure within very few cycles. These findings underscore the essential role of bromide ions in stabilizing reversible Ag electrodeposition. The inherent limitation of Ag-based systems—their dependence on halide ions—motivated our transition to Cu-based system for exploring new material system.

**Figure S22.** Images of the soaking corrosion experiment on the counter electrode Cu foil. (a) Initial state; (b) After 30 minutes; (c) After 24 hours.

The figure above shows the dissolution process over 24 hours, revealing complete dissolution of the 30 nm Cu electrode in electrolyte within 30 minutes, while the DMSO control remained intact. This rapid dissolution confirms the comproportionation reaction between Cu^2+^ and Cu in the electrolyte, generating Cu^+^ species. Importantly, the micrometer-thick Cu foil demonstrated significantly enhanced stability against this self-dissolution effect.


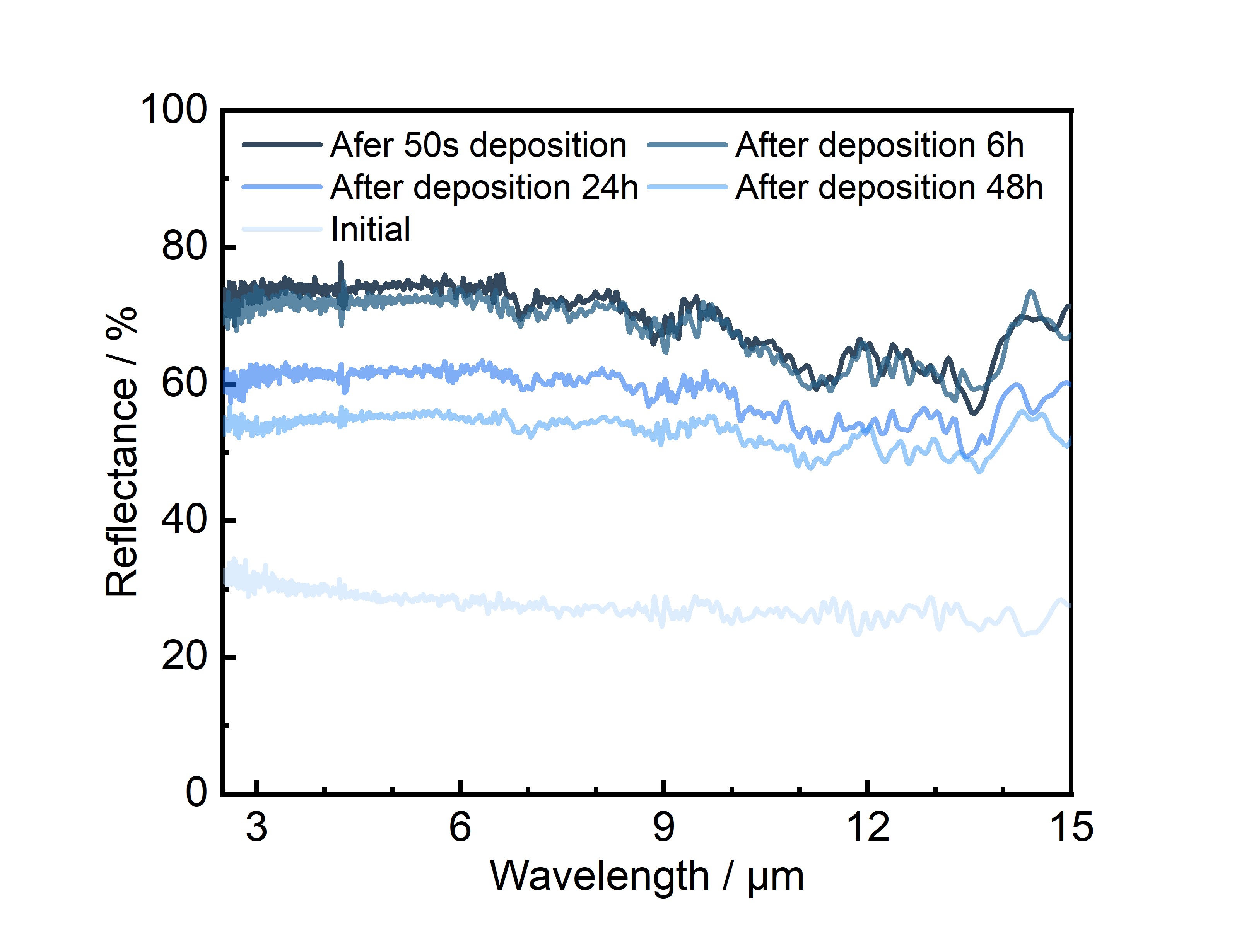


**Figure S23.** IR spectra of Pt-electrode-based copper system device after 6 hours, 24 hours, and 48 hours of open-circuit time.

Open-circuit stability tests showed that the device’s initial reflectance of 30% could be elevated to 70% through 50 s deposition. The system maintained short-term stability with only minor reflectance decay after 6 hours open-circuit. However, prolonged exposure led to gradual corrosion, with reflectance decreasing to 60% (24 h) and 55% (48 h).

**Table S1.** Performance of reported RMEDs for IR radiation modulation.

| Type of electrolyte | Working electrodes | Counter electrodes | Substrate | IR emittance tunabilities（Δε） | Size | Cycle life | Ref |
| --- | --- | --- | --- | --- | --- | --- | --- |
| Cu-PC | Pt-modified Au grids | Copper foil | ZnSe | 0.53（8-12 μm） | / | 500 | ^[5]^ |
| Ag-DMSO | Nanoscopic Pt film | Indium Tin Oxide (ITO) | BaF_2_ / Polypropylene film | 0.71（7.5-13 μm） | 5 cm^2^ | 350 | ^[4]^ |
| Ag-DMSO | Graphene | ITO | BaF_2_ / Polypropylene film | / | / | 4 | ^[6]^ |
| Ag-DMSO | Au grids/Graphene/Pt film | ITO | Polypropylene film | 0.82（7.5-13 μm） | 5 cm^2^ | 350 | ^[7]^ |
| Ag-DMSO | Nanoscopic Pt film | Ag/Pt | Si | 0.48（2.5-25 μm） | 9 cm^2^ | 60 | ^[8]^ |
| Cu-aqueous | Au grids/Graphene/Pt film | Copper foil | Polyethylene film | 0.85（7.5-13 μm） | / | 2500 | ^[9]^ |
| Ag-DMSO | SiC/Pt film | ITO | BaF_2_ | 0.57（3-14 µm） | 6.25 cm^2^ | 100 | ^[10]^ |
| Cu-aqueous | Pt film | Cu | Metastructure substrate | 0.82（3-5 µm）  0.66（8-14 µm） | 100 cm^2^ | 220 | ^[11]^ |

**Table S2.** Average IR transmittance for different substrates.

| substrate | 3-5 µm band | 8-14 µm band |
| --- | --- | --- |
| Silicon | 0.55 | 0.50 |
| BaF_2_ | 0.94 | 0.87 |

**Table S3.** Physical and chemical properties of selected metallic materials ^[12]^.

| Element | Standard reduction potential / V | Resistivity /10^-8^ Ω m | Lattice constant / Å | Crystal structure | Thermal expansion coefficient / 10^-6^×K^-1^ |
| --- | --- | --- | --- | --- | --- |
| Au | 1.70 | 2.05 | 4.08 | FCC | 14.20 |
| Pt | 1.18 | 9.60 | 3.92 | FCC | 8.80 |
| Ir | 1.16 | 4.70 | 3.84 | FCC | 6.40 |
| Ag | 0.80 | 1.47 | 4.09 | FCC | 18.90 |
| Cu | 0.34 | 1.54 | 3.62 | FCC | 16.50 |
| Ni | -0.26 | 6.16 | 3.52 | FCC | 13.40 |
| Cr | -0.74 | 11.80 | 2.91 | BCC | 4.90 |

**Table S4.** Process parameters of electron beam evaporation coating.

| Film | Power1/% | Power2/% | Total power/ kW | Value of Tooling | Rate/ Å s^-1^ | Density/g cm^-3^ |
| --- | --- | --- | --- | --- | --- | --- |
| Pt | 10 | 23 | 5 | 140 | 0.20 | 21.45 |
| Ir | 10 | 23 | 5 | 125 | 0.20 | 22.56 |
| Au | 9 | 18 | 5 | 180 | 0.80 | 19.30 |
| Ag | 5 | 10 | 3 | 146 | 2.00 | 10.49 |
| Cr | 2 | 4 | 3 | 194 | 2.00 | 7.19 |

**References**

[1] K. Sasaki, N. Marinkovic, H. S. Isaacs, R. R. Adzic, *ACS Catalysis.* **2015**, 6, 69.

[2] R. P. Galhenage, K. Xie, W. Diao, J. M. M. Tengco, G. S. Seuser, J. R. Monnier, D. A. Chen, *Physical Chemistry Chemical Physics.* **2015**, 17, 28354.

[3] G. Zhang, X. Zhang, H. Liu, J. Li, Y. Chen, H. Duan, *Advanced Energy Materials.* **2021**, 11, 2003927.

[4] M. Li, D. Liu, H. Cheng, L. Peng, M. Zu, *Science Advances.* **2020**, 6, eaba3494.

[5] B. V. Bergeron, K. C. White, J. L. Boehme, A. H. Gelb, P. B. Joshi, *The Journal of Physical Chemistry C.* **2008**, 112, 832.

[6] M. Li, D. Liu, H. Cheng, L. Peng, M. Zu, *Journal of Materials Chemistry C.* **2020**, 8, 8538.

[7] Y. Rao, J. Dai, C. Sui, Y.-T. Lai, Z. Li, H. Fang, X. Li, W. Li, P.-C. Hsu, *ACS Energy Letters.* **2021**, 6, 3906.

[8] X. Tao, D. Liu, T. Liu, Z. Meng, J. Yu, H. Cheng, *Advanced Functional Materials.* **2022**, 32, 1.

[9] C. Sui, J. Pu, T.-H. Chen, J. Liang, Y.-T. Lai, Y. Rao, R. Wu, Y. Han, K. Wang, X. Li, V. Viswanathan, P.-C. Hsu, *Nature Sustainability.* **2023**, 6, 428.

[10] J. Zhou, Y. Han, D. Ren, Q. Lin, *Journal of Alloys and Compounds.* **2024**, 1002, 175418.

[11] W. Wang, L. Wang, S. Jin, T. Xie, G. Liu, Z. Meng, T. Liu, Y. Cui, H. Zhang, W. Liu, Z. Gao, B. Wang, L. Wang, *ACS Energy Letters.* **2025**, 10, 3231.

[12] A. L.-S. Eh, J. Chen, X. Zhou, J.-H. Ciou, P. S. Lee, *ACS Energy Letters.* **2021**, 6, 4328.
